# Novel thiazole-based cyanoacrylamide derivatives: DNA cleavage, DNA/BSA binding properties and their anticancer behaviour against colon and breast cancer cells

**DOI:** 10.1186/s13065-024-01284-2

**Published:** 2024-09-20

**Authors:** Karim Barakat, Mohamed A. Ragheb, Marwa H. Soliman, Amr M. Abdelmoniem, Ismail A. Abdelhamid

**Affiliations:** 1 Department of Chemistry (Biochemistry Division), Faculty of Science, Cairo University, Giza, 12613 Egypt; 2 Department of Chemistry, Faculty of Science, Cairo University, Giza, 12613 Egypt

**Keywords:** Cyanoacrylamide, DNA photocleavage, DNA/BSA binding, Cytotoxicity, HCT116 cells, MDA-MB-231 cells, Photodynamic therapy

## Abstract

A novel series of 2-cyano-3-(pyrazol-4-yl)-*N*-(thiazol-2-yl)acrylamide derivatives (**3a**–**f**) were synthesized using Knoevenagel condensation and characterized using various spectral tools. The weak nuclease activity of compounds **(3a**–**f)** against pBR322 plasmid DNA was greatly enhanced by irradiation at 365 nm. Compounds **3b** and **3c**, incorporating thienyl and pyridyl moieties, respectively, exhibited the utmost nuclease activity in degrading pBR322 plasmid DNA through singlet oxygen and superoxide free radicals’ species. Furthermore, compounds **3b** and **3c** affinities towards calf thymus DNA (CT-DNA) and bovine serum albumin (BSA) were investigated using UV–Vis and fluorescence spectroscopic analysis. They revealed good binding characteristics towards CT-DNA with K_b_ values of 6.68 × 10^4^ M^−1^ and 1.19 × 10^4^ M^−1^ for **3b** and **3c**, respectively. In addition, compounds **3b** and **3c** ability to release free radicals on radiation were targeted to be used as cytotoxic compounds in vitro for colon (HCT116) and breast cancer (MDA-MB-231) cells. A significant reduction in the cell viability on illumination at 365 nm was observed, with IC_50_ values of 23 and 25 µM against HCT116 cells, and 30 and 9 µM against MDA-MB-231 cells for compounds **3b** and **3c**, respectively. In conclusion, compounds **3b** and **3c** exhibited remarkable DNA cleavage and cytotoxic activity on illumination at 365 nm which might be associated with free radicals’ production in addition to having a good affinity for interacting with CT-DNA and BSA.

**Graphical Abstract:**

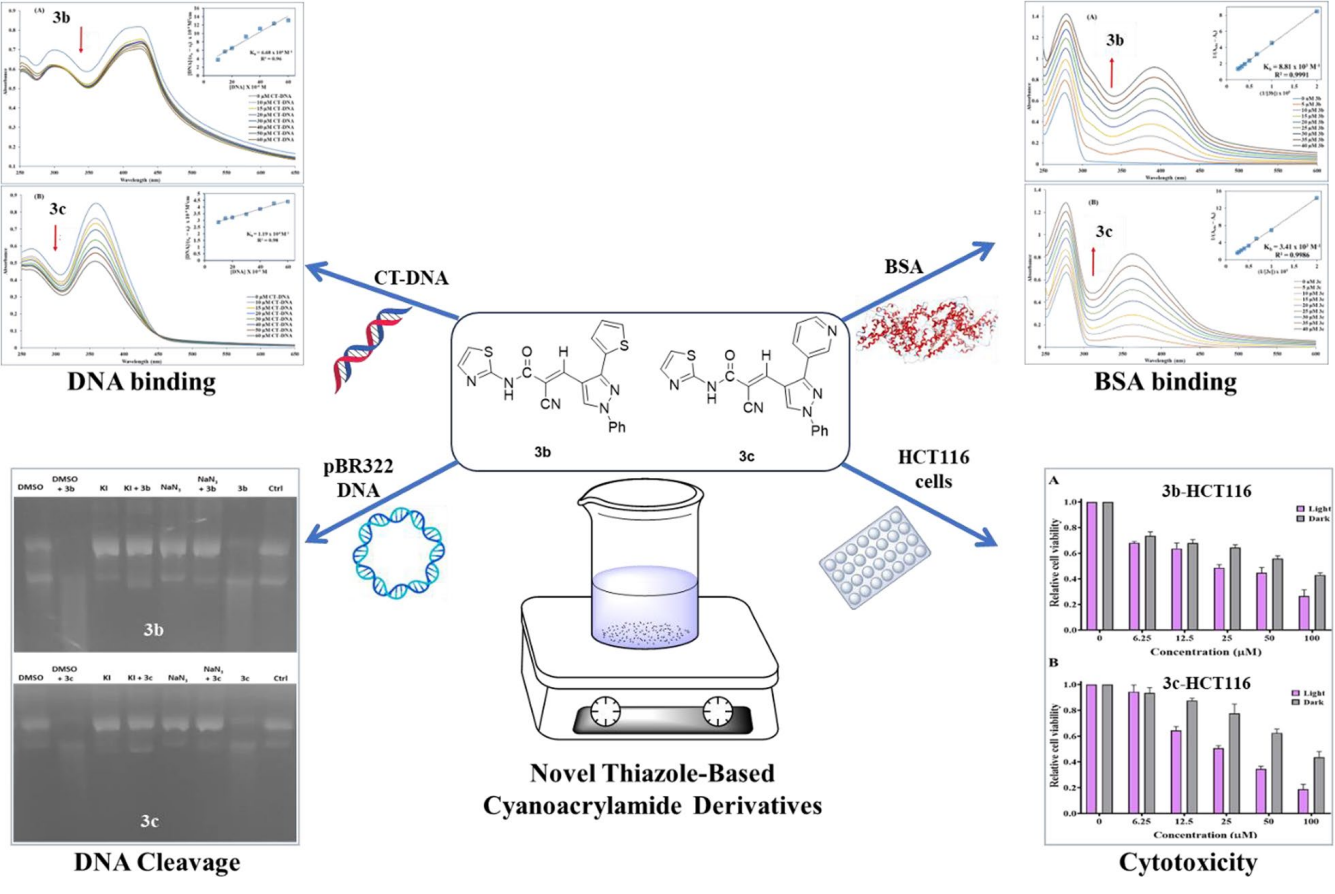

**Supplementary Information:**

The online version contains supplementary material available at 10.1186/s13065-024-01284-2.

## Introduction

Over the previous decade, there has been rapid growth in research areas that focus on the synthesis of various molecules that exhibit biological activity and can be utilized for various pharmaceutical purposes [1–4]. The promising category of heterocyclic organic compounds exhibits a broad spectrum of physical, chemical, and biological characteristics that could fulfill these intentions [5–8]. For example, nitrogen-containing heterocycles are one of the unique compounds well-known for their engagement in medicinal sciences [9, 10]. Pyrazole is a heterocyclic compound whose chemical structure comprises a five-membered ring embracing two adjacent nitrogen atoms [11] and is renowned for its uses as a pharmaceutical agent utilizing its anti-inflammatory [12], anticancer [13], antimicrobial [14], and various therapeutic effects [15]. Thiazole ring is a nitrogen/sulfur atoms five-membered ring [16], which is acquainted for its anti-inflammatory [17], antiviral [18], treatment of Alzheimer's disease [19], and antibacterial [20], besides its utilization in cancer therapy, in which some of its derivatives exhibited a potential effect, particularly in the management of colon and breast cancer [21–25]. Furthermore, cyanoacrylamide moiety is appreciated for its anticancer activity which has been reported for certain derivatives in the treatment of breast and colon cancer [26–29], antimicrobial [30], antiviral [31], and diversity of biological activities, for instance, DNA fragmentation [32], kinase/anhydrase inhibitors [33], and antioxidant activity [34].

Numerous scientific fields, including biochemistry, pharmaceutical chemistry, and cancer therapy, have focused on exploring the interaction between small molecules and DNA [35–37]. Since DNA is a key pharmacological goal and how a drug binds to DNA determines how successful it is, this is the initial step in developing a new class of therapeutic medicines. In general, a series of covalent and non-covalent interactions enhance the chemotherapeutic agent binding to DNA [38]. In malignant cells, these interactions give rise to DNA damage and hinder replication and/or transcription, which ultimately leads to cell death [39].

Serum albumins have a variety of physiological roles and represent about 55% of the proteins in the blood. They serve as a carrier protein for a wide range of endogenous and exogenous molecules, including fatty acids, hormones, and several medications. Because of its affordability, high degree of resemblance to human serum albumin, and ease of availability, BSA is considered an ideal model in the study of drug-protein interactions. Therefore, it is crucial to investigate the interaction of small molecules with BSA to provide an extra benefit for using them as possible chemotherapeutic agents [40, 41].

In continuation of our interest in the synthesis of bioactive heterocycles [42–48] herein, novel thiazole-related cyanoacrylamide derivatives were synthesized via Knoevenagel condensation. The cleavage activity of the synthesized compounds against pBR322 plasmid DNA was assessed using agarose gel electrophoresis with investigating the potential mechanism involved. Besides, the interaction of the potent compounds with different biological molecules such as CT-DNA and BSA using fluorescence and UV–visible spectroscopy was also investigated to understand the mode of binding and the affinity of these compounds towards those crucial biomolecules. Furthermore, the cytotoxic effect of the active cyanoacrylamide against human cancer cell lines (HCT116 and MDA-MB-231 cells) was examined to explore their ability to be used as chemotherapeutic agents.

## Experimental

### Materials

2-Aminothiazole was bought from HIMEDIA (India), and all pyrazole aldehydes used were obtained as previously [49]. Solvents of analytical grade were purchased from Sigma-Aldrich or Merck. Calf-thymus DNA (CT-DNA, CAS 73049-39-5), agarose (molecular biology grade, CAS 9012-36-6), doxorubicin (CAS 25316-40-9), 3-(4,5-dimethylthiazol-2-yl)-2,5-tetrazolium bromide (MTT, CAS 298-93-1), and ethidium bromide (EtBr, CAS 1239-45-8) were procured from Sigma-Aldrich (USA). Bovine serum albumin (BSA) was purchased from BioBasic Inc. (Canada). Supercoiled pBR322 DNA was obtained from SibEnzyme Ltd. (Russia). DMEM, fetal bovine serum, and penicillin/streptomycin were provided by Gibco, Thermo Fisher Scientific Inc.

### Physical measurements

Melting points were measured with a Stuart melting point apparatus and are uncorrected. The IR spectra were recorded using a FTIR Bruker–vector 22 spectrophotometer as KBr pellets. The ^1^H NMR spectra were recorded in dimethyl sulfoxide (DMSO)–*d*_6_ as a solvent on Varian Gemini NMR spectrometer at 300 MHz or Bruker AVS NMR spectrometer at 500 MHz using TMS as the internal standard. Chemical shifts are reported as δ values in ppm. Mass spectra were recorded with a Shimadzu GCMS–QP–1000 EX mass spectrometer in EI (70 eV) model. The elemental analyses were performed at the Microanalytical centre, Cairo University. Electronic absorption spectra were recorded using a Shimadzu UV-3101 spectrophotometer in the range of 200–800 nm. Fluorescence spectra were recorded on a spectrofluorometer Jasco FP-6200, Japan.

### General procedure for the synthesis of thiazole-based cyanoacrylamide derivatives

A mixture of 2-cyano-*N*-(thiazol-2-yl)acetamide (**1**) (167 mg, 10 mmol) and the corresponding aldehydes **2a**–**f** (10 mmol) was heated at reflux for 3 h in absolute EtOH (10 mL) in the presence of piperidine (0.2 mL, 2 mmol) as a basic catalyst. The formed solid product was then filtered, washed with ethanol, dried, and crystallized from EtOH/dioxane (5:1, v/v) to give 2-cyanoacrylamide derivatives **3a**–**f**.

#### 2-Cyano-3-(1,3-diphenyl-1*H*-pyrazol-5-yl)-*N*-(thiazol-2-yl)acrylamide (3a)


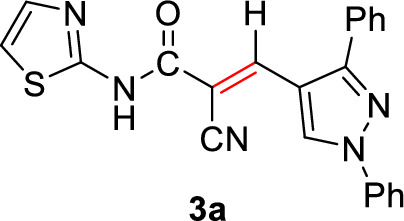


Pale yellow solid (365 mg, 92%); Mp 265–267°C; IR (KBr): $\overline{\nu }$ 3325 (N*H*), 2307 (C≡N), 1697 (C=O amide), 1636 (C=C) cm^−1^; ^1^H NMR (500 MHz, DMSO-*d*_6_; Fig. S1): δ 7.16 (d, *J* = 4.4 Hz, 1H, Ar–*H*), 7.43 (t, *J* = 7.4 Hz, 1H, Ar–*H*), 7.48 (d, *J* = 4.2 Hz, 1H, Ar–*H*), 7.52–7.59 (m, 6H, Ar–*H*), 7.66 (d, *J* = 7.9 Hz, 2H, Ar–*H*), 7.91 (d, *J* = 8.4 Hz, 2H, Ar–*H*), 8.23 (s, 1H, vinylic H), 9.17 (s, 1H, pyrazolyl-*H*5), 13.18 (s, 1H, N*H*) ppm. MS (EI, 70 eV): *m/z* (%) 397]M^+^[; Anal. Calcd for C_22_H_15_N_5_OS: C, 66.48; H, 3.80; N, 17.62; S, 8.07. Found: C, 66.31; H, 3.65; N, 17.44; S, 8.01.

#### 2-Cyano-3-(1-phenyl-3-(thiophen-2-yl)-1*H*-pyrazol-4-yl)-*N*-(thiazol-2-yl)acrylamide (3b)


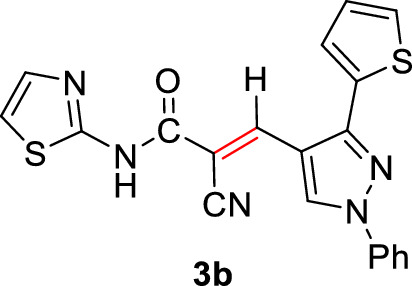


Pale yellow solid (359 mg, 89%); Mp 252–256°C; IR (KBr): $\overline{\nu }$ 3294 (N*H*), 2214 (CN), 1662 (C=O amide) cm^−1^; ^1^H NMR (500 MHz, DMSO-*d*_6_; Fig. S2): δ 7.19 (t, *J* = 5.1 Hz, 1H, Ar–*H*), 7.28 (d, *J* = 4.5 Hz, 1H, Ar–*H*), 7.40–7.63 (m, 5H, Ar–*H*), 7.78 (d, *J* = 5.4 Hz, 1H, Ar–*H*), 7.89 (d, *J* = 7.9 Hz, 2H, Ar–*H*), 8.41 (s, 1H, vinylic C*H*), 9.17 (s, 1H, pyrazolyl-*H*5), 13.16 (s, 1H, N*H*) ppm; MS (EI, 70 eV): *m/z* (%) 403]M^+^[; Anal. Calcd for C_20_H_13_N_5_OS_2_: C, 59.54; H, 3.25; N, 17.36; S, 15.89. Found: C, 59.32; H, 3.07; N, 17.21; S, 15.79.

#### 2-Cyano-3-(1-phenyl-3-(pyridin-3-yl)-1*H*-pyrazol-4-yl)-*N*-(thiazol-2-yl)acrylamide (3c)


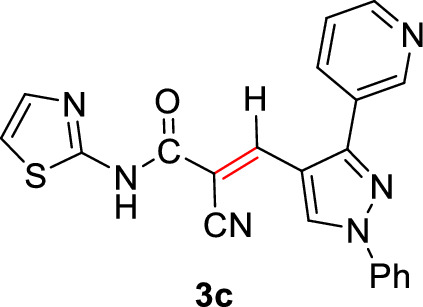


Pale yellow solid (354 mg, 89%); Mp 250–252°C; IR (KBr): $\overline{\nu }$ 3292 (N*H*), 2214 (CN), 1667 (C=O amide) cm^−1^; ^1^H NMR (300 MHz, DMSO-*d*_6_; Fig. S3): δ 7.20 (d, *J* = 4.0 Hz, 1H, Ar–*H*), 7.40–7.71 (m, 5H, Ar–*H*), 7.95 (d, *J* = 8.2 Hz, 2H, Ar–*H*), 8.14 (dd, *J* = 7.9, 2.1 Hz, 1H, Ar–*H*), 8.25 (s, 1H, vinylic C*H*), 8.75 (d, *J* = 4.8 Hz, 1H, Ar–*H*), 8.92 (s, 1H, pyridin-*H3*), 9.23 (s, 1H, pyrazolyl-*H*5), 13.11 (s, 1H, N*H*) ppm; ^13^C NMR (75 MHz, DMSO-*d*_6_; Fig. S4): δ 112.9, 115.1, 117.2, 119.7, 124.1, 127.1, 128.2, 129.5, 129.9, 130.4, 131.6, 136.5, 138.6, 141.4, 149.2, 150.2, 151.9, 157.8, 164.7 ppm; MS (EI, 70 eV): *m/z* (%) 398]M^+^[; Anal. Calcd for C_21_H_14_N_6_OS: C, 63.30; H, 3.54; N, 21.09; S, 8.05. Found: C, 63.17; H, 3.43; N, 21.03; S, 8.12.

#### 2-Cyano-3-(1-phenyl-3-(*p*-tolyl)-1*H*-pyrazol-4-yl)-*N*-(thiazol-2-yl)acrylamide (3d)


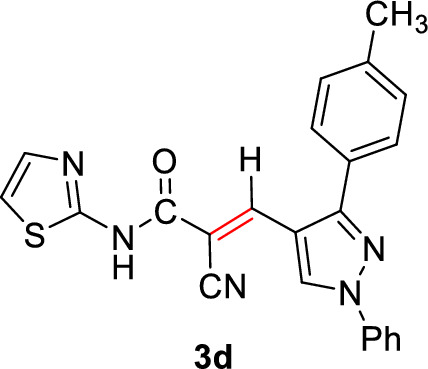


Pale yellow solid (308 mg, 75%); Mp 255–257°C; IR (KBr): $\overline{\nu }$ 3232 (N*H*), 2216 (CN), 1674 (C=O) cm^−1^; ^1^H NMR (300 MHz, DMSO-*d*_6_; Fig. S5): δ 2.41 (s, 3H, C*H*_3_), 7.18 (d, *J* = 4.0 Hz, 1H, Ar–*H*), 7.40 (d, *J* = 8.1 Hz, 2H, Ar–*H*), 7.48 (dd, *J* = 10.4, 5.7 Hz, 2H, Ar–*H*), 7.54–7.70 (m, 4H, Ar–*H*), 7.92 (d, *J* = 7.7 Hz, 2H, Ar–*H*), 8.26 (s, 1H, vinylic C*H*), 9.17 (s, 1H, pyrazolyl-*H*5), 13.17 (s, 1H, N*H*) ppm; MS (EI, 70 eV): *m/z* (%) 411]M^+^[; Anal. Calcd for C_23_H_17_N_5_OS: C, 67.14; H, 4.16; N, 17.02; S, 7.79. Found: C, 67.04; H, 4.09; N, 17.14; S, 7.62.

#### 2-Cyano-3-(3-(4-methoxyphenyl)-1-phenyl-1*H*-pyrazol-4-yl)-*N*-(thiazol-2-yl) acrylamide (3e)


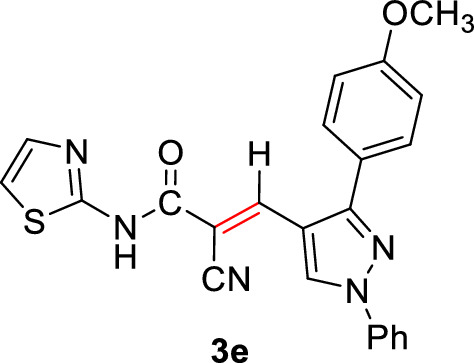


Pale yellow solid (342 mg, 80%); Mp 252–254°C; IR (KBr): $\overline{\nu }$ 3236 (N*H*), 2214 (CN), 1667 (C=O) cm^−1^; ^1^H NMR (300 MHz, DMSO-*d*_6_; Fig. S6): δ 3.85 (s, 3H, OC*H*_3_), 7.16 (m, 3H, Ar–*H*), 7.48 (dd, *J* = 12.6, 5.8 Hz, 2H, Ar–*H*), 7.54–7.67 (m, 4H, Ar–*H*), 7.92 (d, *J* = 8.0 Hz, 2H, Ar–*H*), 8.26 (s, 1H, vinylic C*H*), 9.16 (s, 1H, pyrazolyl-*H*5), 12.81 (s, 1H, N*H*) ppm; MS (EI, 70 eV): *m/z* (%) 427]M^+^[; Anal. Calcd for C_23_H_17_N_5_OS: C, 67.14; H, 4.16; N, 17.02; S, 7.79. Found: C, 67.04; H, 4.09; N, 17.14; S, 7.62.

#### 3-(3-(4-Chlorophenyl)-1-phenyl-1*H*-pyrazol-4-yl)-2-cyano-*N*-(thiazol-2-yl)acrylamide (3f)


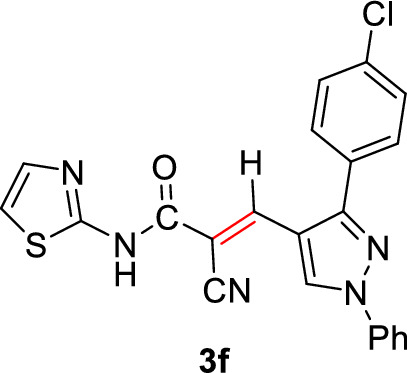


Pale yellow solid (358 mg, 83%); Mp 270–272°C; IR (KBr): $\overline{\nu }$ 3240 (N*H*), 2215 (CN), 1674 (C=O) cm^−1^; ^1^H NMR (300 MHz, DMSO-*d*_6_; Fig. S7): δ 7.19 (d, *J* = 4.0 Hz, 1H, Ar–*H*), 7.42–7.54 (m, 2H, Ar–*H*), 7.56–7.77 (m, 6H, Ar–*H*), 7.93 (d, *J* = 8.5 Hz, 2H, Ar–*H*), 8.25 (s, 1H, vinylic C*H*), 9.19 (s, 1H, pyrazolyl-*H*5), 13.16 (s, 1H, N*H*) ppm; MS (EI, 70 eV): *m/z* (%) 431]M^+^[; Anal. Calcd for C_22_H_14_ClN_5_OS: C, 61.18; H, 3.27; Cl, 8.21; N, 16.22; S, 7.42. Found: C, 61.11; H, 3.21; Cl, 8.11; N, 16.07; S, 7.19.

### DNA cleavage studies

Using agarose gel electrophoresis, the DNA cleavage studies of the synthesized compounds were conducted in the absence and presence of irradiation at 365 nm. The compounds were solubilized in DMSO and diluted to a final concentration (200 μM) when mixed with Tris/NaCl buffer (pH 7.1) containing pBR322 plasmid DNA (0.3 μg), where the final DMSO percentage was 5%. Then, the mixture was incubated at 37°C for 30 min for dark conditions and followed by illumination for irradiated conditions using UV-A light at 365 nm for 60 min at room temperature. Subsequently, the solutions were mixed with loading buffer (10 mM Tris–HCl (pH 7.6), 0.03% bromophenol blue, 0.03% xylene cyanol FF, 60% glycerol, and 60 mM EDTA) and loaded on the agarose-gel (1%, w/v), comprised of TBE buffer (Tris–HCl, boric acid, and EDTA) treated with (0.5 μg/ml) EtBr. Samples were run in the previous system for 2 h at 75 V and the gel images were acquired by UV trans-illuminator at the end of the process. ImageJ software was used to assess the percentage of cleavage fragments [50]. To conduct mechanistic pBR322 DNA photo-cleavage investigations of the selected compounds, 200 μM of the **3b** and **3c** were independently mixed with pBR322 plasmid DNA in the presence and absence of several radical scavengers, such as NaN_3_, KI, and DMSO, as previously reported [50].

### DNA binding studies

CT-DNA stock solution was prepared by dissolving CT-DNA in Tris/NaCl buffer (pH 7.1). Then, it was stored at 4°C and was manipulated within 4 days of preparation. The concentration was estimated by applying the equation $C= \frac{{A}_{260 nm}}{\varepsilon \times \text{ b}}$ where C is the concentration of DNA solution, A is the absorbance at wavelength 260 nm, b is the path length and ε is the molar extinction coefficient of DNA that equals 6600 M^−1^ cm^−1^. The purity also was calculated using the absorbance ratio at wavelength 260 and 280 respectively, which was 1.8 indicating free DNA from protein contamination. Stock solutions of compounds **3b** and **3c** were prepared by dissolving in DMSO.

#### DNA UV–Visible absorption spectrophotometric studies

The interaction between the selected compounds (**3b** and **3c**) and CT-DNA was assessed through the spectrophotometric titration method [50]. In brief, a fixed concentration of compounds (25 μM) was mixed with an accumulative addition of CT-DNA (0–60 μM) at room temperature with a constant DMSO percentage of 5%. After 5 min from each addition, the UV–Vis absorption spectrum (250–650 nm) was recorded. The hyper/hypochromic percentage change at the characteristic peak of **3b** and **3c** was assessed using the formula: $H\%= \frac{{A}_{free}- {A}_{bound}}{{A}_{bound}}\times 100$. While the binding constant (K_b_) of **3b** and **3c** was estimated using Wolfe-Shimmer Equation [51]:

$$\frac{[DNA]}{({\upvarepsilon }_{a}- {\upvarepsilon }_{f})}=\frac{[DNA]}{({\varepsilon }_{b}- {\varepsilon }_{f})}+ \frac{1}{{K}_{b}({\varepsilon }_{b}- {\varepsilon }_{f})}$$

where [DNA] denotes CT-DNA concentration, ε_a_, ε_f_ and ε_b_ stands for the apparent absorption coefficient, the extinction coefficient of the unbound compound, and the extinction coefficient when fully bound to DNA respectively. K_b_ is estimated from [DNA] / (ε_a_ − ε_f_) versus [DNA] plots by computation of the ratio of slope to intercept. Standard Gibbs free energy (ΔG°) was calculated using the equation ΔG° =  − RTlnK_b_ (where T is the temperature, 298 K; R is the universal gas constant, 8.314 J K^−1^ mol^−1^).

#### The Ethidium Bromide quenching assay

EtBr is a conventional intercalator whose fluorescence intensity intensifies in response to CT-DNA interaction. Solution of CT-DNA-EtBr with final concentrations of EtBr (20 µM) and CT-DNA (100 µM) in Tris–HCl buffer solution (pH = 7.1) was prepared, then compounds were solubilized in DMSO, added to the prepared solution to attain a range of concentration (0–160 µM) and (0–80 µM) for **3b** and **3c**, respectively. Then, the mixtures were incubated 3 min after each addition and the emission spectra (540–710 nm) were recorded using an excitation wavelength of 520 nm.

### BSA binding studies

BSA was dissolved in Tris/NaCl buffer (pH 7.1) to prepare the BSA stock solution, which was then kept at 4°C and utilized within a week after preparation [52, 53]. The concentration was evaluated by applying the equation $C= \frac{{A}_{280 nm}}{\varepsilon \times \text{ b}}$ where C is the concentration of protein solution, A is the absorbance at wavelength 280 nm, b is the path length and ε is molar extinction coefficient of protein that equals 44,300 M^−1^ cm^−1^.

#### BSA UV–Visible absorption spectrophotometric studies

The interaction was evaluated through dilution of BSA to 15 μM in Tris/NaCl (pH 7.1) during the spectrophotometric titration. After that, the compounds dissolved in DMSO were added with a range of increasing concentration (0–40 μM), where the final DMSO percentage is 5%. UV–Vis absorption spectrum measurement for each concentration was assessed by Shimadzu UV-3101 PC NIR spectrophotometer after 3 min of each addition. The K_b_ for compounds **3b** and **3c** was calculated using equation [54]:

$$\frac{1}{{\text{A}}_{obs}- {\text{A}}_{0}}=\frac{1}{{A}_{c}- {A}_{0}}+ \frac{1}{{K}_{b}\left({A}_{c}- {A}_{0}\right)[\text{compound }]}$$

where A_0,_ A_obs_ and A_c_ are absorbance of solution in the absence, various concentrations, and saturation of the compound at the characteristic peak, respectively. Where K_b_ is estimated from the plot of $\frac{1}{{\text{A}}_{obs}- {\text{A}}_{0}}$ versus $\frac{1}{[Drug]}$. Hence, K_b_ is evaluated from the ratio between intercept and slope.

#### Tryptophan quenching assay with BSA

BSA (15 µM) solution was prepared in Tris/HCl buffer solution (pH = 7.1) and mixed with serial concentrations of compounds **3b** and **3c** (0–40 µM), achieving 5% final concentration of DMSO. Following 3 min incubation after each addition, fluorescence quenching spectra were recorded from 300 to 450 nm using a spectrofluorometer with an excitation wavelength of 289 nm.

### Cell viability assay

The cytotoxicity of **3b** and **3c** against MDA-MB-231 and HCT116 cell lines, with and without illumination, was assessed using the MTT test [55]. Two human cancer cell lines, including MDA-MB-231 and HCT116 cell lines, were purchased from the National Research Centre, Dokki, Cairo, Egypt. Briefly, 96-well plates were seeded with cells (5 × 10^3^ cells/well) and left overnight. The compounds dissolved in DMSO were then applied to the cells following the serial dilution procedure in the range of 0–100 µM. The cells were then incubated for an additional 20 h. Subsequently, the cells were exposed to UV light for 20 min at a distance of 10 cm. To study the effects of the illumination, a control plate was created under the same circumstances but without illumination. The two plates were incubated for a further 20 h. After that, PBS was used to wash the cells and MTT solution (0.5 mg/ml) was added. To dissolve the generated formazan crystals, the solution was replaced with 100 µl of DMSO per well after 4 h of incubation. An ELISA reader operating at 492 nm was used to measure the absorbance after 15 min of plate shaking. By relating the absorbance of treated and negative control cells, the relative cell viability was calculated. GraphPad Prism software was used to calculate the half inhibitory concentration (IC_50_). Doxorubicin (0–100 μM) was utilized as a positive cytotoxicity control in the usual dark environment, while the vehicle (DMSO, 0.1%) was used as a negative control.

## Results and discussion

### Synthetic chemistry

**Scheme 1.**
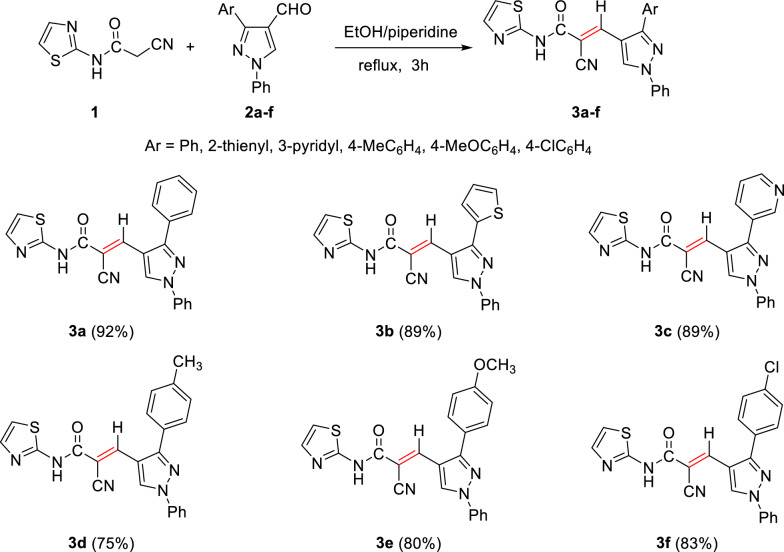
Knoevenagel condensation reaction of 2-cyano-*N*-(thiazol-2-yl)acetamide (**1**) with different aldehydes **2a**–**f**

2-Cyano-*N*-(thiazol-2-yl)acetamide (**1**) was obtained in high yields and purity following the method reported by us [28, 56] and others earlier [26]. Knoevenagel condensation of 2-cyano-*N*-(thiazol-2-yl)acetamide (**1**) with aromatic aldehydes **2a**–**f** in the presence of piperidine as a basic catalyst affords the corresponding cyanoacrylamides incorporating thiazole and pyrazole moieties **3a**–**f** in very good yields (75–92%) (Scheme 1). The constitutions of the obtained products were confirmed based on spectral data. Thus, ^1^H NMR of compound **3c** as a representative example indicated four singlet signals at 8.25, 8.92, 9.23, and 13.11 corresponding to vinylic C*H*, pyridin-*H3*, pyrazole-*H*5, and N*H*, respectively. The aromatic protons appear at their expected positions. The ^13^C NMR featured 19 signals corresponding to 19 different signals.

### DNA cleavage

According to Modi et al., pyrazole derivatives have been reported to have DNA cleavage activity, where all compounds incorporating pyrazole moiety can generate detectable plasmid DNA cleavage at 100 μM [57]. Furthermore, thiazole hybrids appeared to have promising effects on DNA cleavage activity [58]. DNA cleavage activity of the synthesized compounds bearing pyrazole and thiazole rings in combination with cyanoacrylamide moiety was estimated chemically using gel electrophoresis. When plasmid DNA is mixed with the cleavage agent and loaded for gel electrophoresis, the supercoiled form exhibits the fastest migration (Form I), followed by the nicked circular (Form II) owing to a break in one strand which moves more slowly as it becomes loose. Mutual cleavage of the two strands, on the other hand, produces a linear form (Form III), which has a moderate migration speed [59, 60].

#### Chemically induced DNA cleavage by gel electrophoresis

**Fig. 1:**
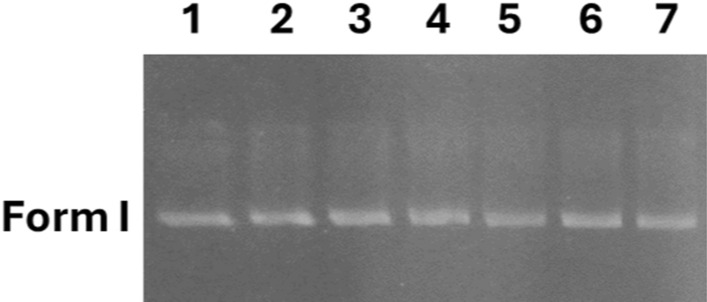
Agarose gel electrophoresis pattern of pBR322 DNA (0.3 μg) cleavage by compounds **3a**–**f** (200 μM) incubated in the dark at 37°C for 30 min. Lane 1: DNA control; Lane 2: DNA + **3a**; Lane 3: DNA + **3b**; Lane 4: DNA + **3c**; Lane 5; DNA + **3d**; Lane 6: DNA + **3e**; Lane 7: DNA + **3f**

Compounds **3a**–**f** (200 μM) displayed insignificant cleavage activity with pBR322 DNA (0.3 μg) in Tris/NaCl (5 mM Tris–HCl/50 mM NaCl (pH 7.1)) buffer containing 5% DMSO, as illustrated in Fig. 1 and Fig. S8.

#### Photo-induced DNA cleavage by gel electrophoresis

**Fig. 2:**
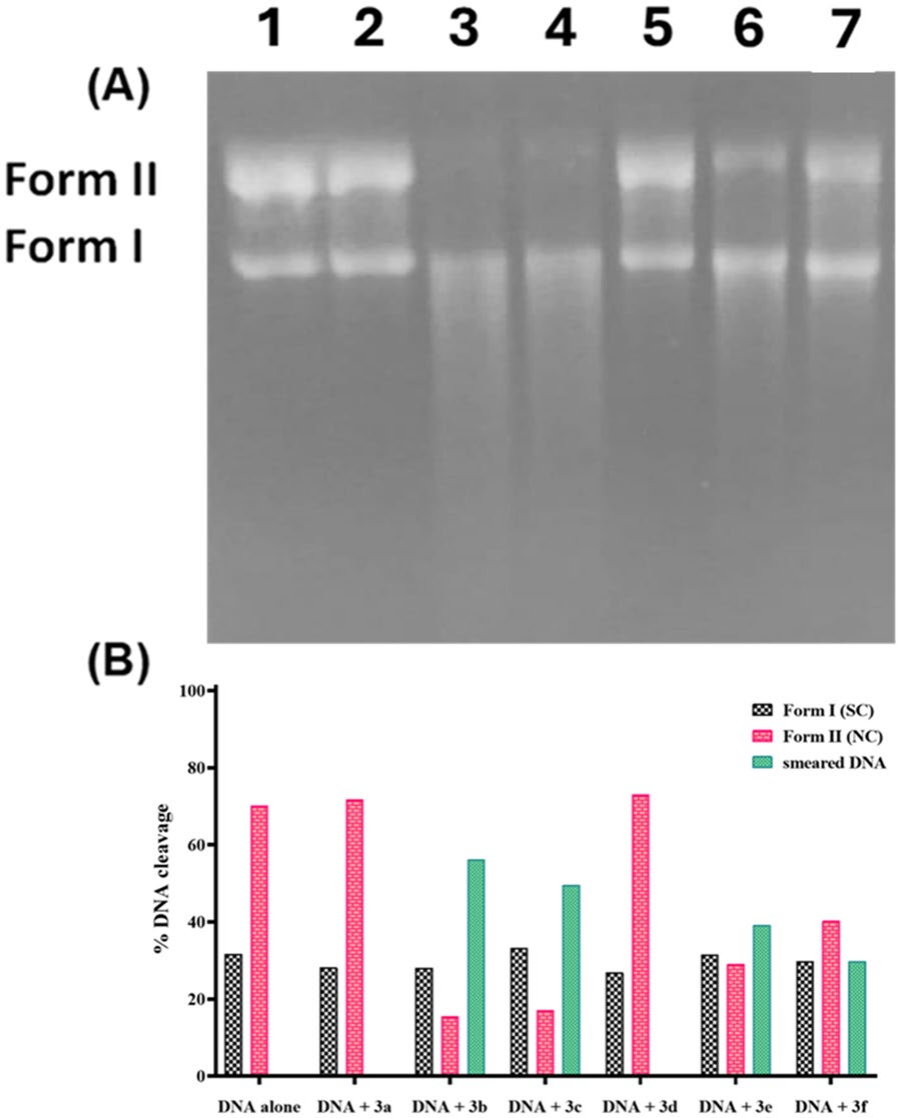
Agarose gel electrophoresis pattern of pBR322 DNA (0.3 μg) cleavage by compounds **3a**–**f** (200 μM) incubated in the dark at 37°C for 30 min followed by irradiation at 365 nm for 60 min (**A**). Lane 1: DNA control; Lane 2: DNA + **3a**; Lane 3: DNA + **3b**; Lane 4: DNA + **3c**; Lane 5; DNA + **3d**; Lane 6: DNA + **3e**; Lane 7: DNA + **3f**. Histogram illustrated the DNA cleavage percentage for each compound (**B**)

The DNA photocleavage activity of these compounds was examined under the same conditions, in addition to exposing them along with pBR322 DNA for an incubation time of 30 min at 37°C followed by irradiation at 365 nm for 60 min. Figure 2 and Fig. S9 depicted that compounds **3a** and **3d** exhibited non-detectable DNA cleavage, while the rest showed remarkable photocleavage activity uniquely **3b** and **3c**.

#### Structure–activity relationship (SAR)

**Fig. 3:**
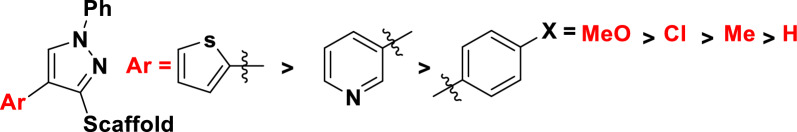
The photocleavage activity of cyanoacrylamide derivatives structure–activity

In general, the stability of aromatic compounds dictates how applied UV light will affect them, determining their ability to withstand structural changes and the production of free radicals. Mainly, the benzene ring has reputable stability owing to its high resonance energy, unlike the heterocyclic pyridine and thiophene rings which have lower resonance energies. This stability arrangement may be responsible for their susceptibility to photochemical reactions and their capability of producing free radicals culminating in their DNA cleavage potentials. Furthermore, the ring strain has been attributed to the enhancement of thiophene and pyridine's reactivity propensity toward photochemical processes, and the creation of distinct photoproducts or reaction pathways missing in less strained aromatic compounds [61–66]. As a result, it was found that the newly synthesized compounds containing heterocyclic moieties had superior photo-cleavage activity than those substituted with aromatic moieties. As illustrated in Fig. 3, thienyl (5-membered ring)-substituted derivative **3b** was more effective than pyridinyl (6-membered ring)-substituted one **3c**. Meanwhile, the aromatic-substituted derivatives demonstrated that the electron-withdrawing chloro-substituted derivative **3f** displayed a lower activity than electron-donating methoxy-substituted compound **3e**. But more effective than methyl-substituted derivative **3d**. Notably, the substituted aromatic derivatives generally displayed higher activity than the unsubstituted aromatic derivative **3a**.

##### Mechanistic pBR322 DNA photo-cleavage studies

**Fig. 4:**
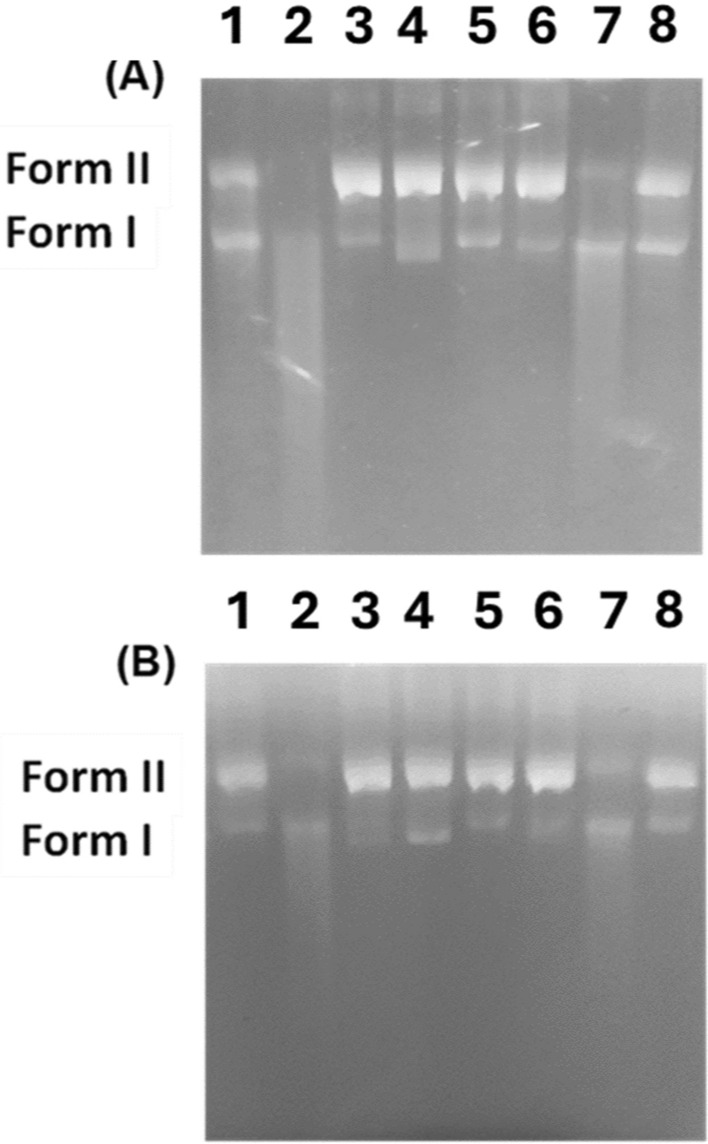
Agarose gel electrophoresis pattern of pBR322 DNA (0.3 μg) cleavage by **3b** (200 μM) (**A**) and **3c** (200 μM) (**B**) with different free radical scavengers incubated in the dark at 37°C for 30 min followed by irradiation at 365 nm for 60 min. DNA. Lane 1: DNA + DMSO (200 mM); Lane 2: DNA + **3b**/**3c** + DMSO (200 mM); Lane 3: DNA + KI (200 mM); Lane 4: DNA + **3b**/**3c** + KI (200 mM); Lane 5; DNA + NaN_3_ (200 mM); Lane 6: DNA + **3b**/**3c** + NaN_3_ (200 mM); Lane 7: DNA + **3b**/**3c**; Lane 8: DNA control

To determine their mechanistic effect, compounds **3b** and **3c** were chosen for further investigation. Different free radical scavengers such as DMSO (hydroxyl radical scavenger), KI (superoxide radical scavenger), and NaN_3_ (singlet oxygen scavenger) were added along with the irradiated incubation of pBR322 DNA with compounds **3b** and **3c**. The scavenger’s addition is to elucidate the photocleavage mechanism of these compounds. The outcomes from Fig. 4 and Fig. S10 have shown total hindrance of compounds **3b** and **3c** activity in the presence of NaN_3_ and KI revealing that the mechanistic action of these compounds is through singlet oxygen and superoxide free radicals’ species. While the presence of DMSO did not influence the photocleavage activity of the compounds so hydroxyl radicals are not implicated in the compound’s activity. Consequently, **3b** and **3c** were selected for additional investigation utilising different conformation of DNA (CT-DNA) and BSA.

### DNA binding studies

#### UV–Vis measurements

**Fig. 5:**
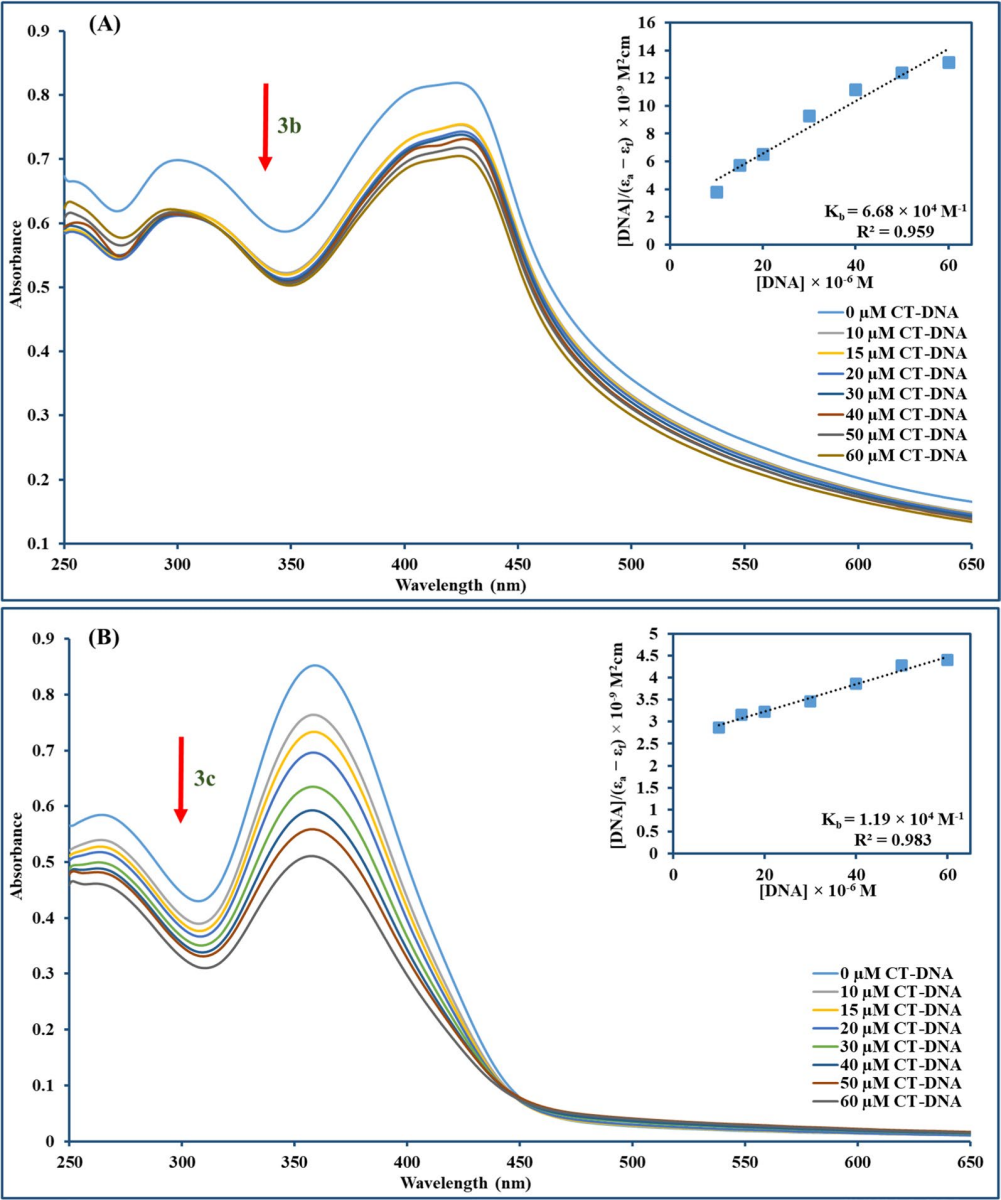
Absorption spectra of **3b** (**A**) and **3c** (**B**) show a hypochromic effect for both compounds denoted by the arrow direction along with increasing CT-DNA concentration (0–60 µM). The incorporated graph exemplifies the plot used for binding constant (K_b_) computation using Wolfe–Shimmer equation

Electronic absorption titration is the technique most frequently used for investigating the interaction between a small molecule and DNA through monitoring the changes in absorption spectra (position or absorbance of the bands) resulting from the interaction between the examined compounds and DNA [67]. Compounds could interact with DNA non-covalently through different forms, including intercalation between stacked DNA base pairs, groove binding, or electrostatic contact with the negatively charged backbone of DNA [68–70]. As depicted in Fig. 5, UV–Vis spectra of compounds **3b** and **3c** exhibited distinctive peaks at 423 nm and 359 nm with a hypochromism (~ 14% and ~ 40%) after adding increasing concentrations of CT-DNA to **3b** and **3c**, respectively, with slight blue shift (~ 1 nm) for both compounds. Compound-DNA complex stability was measured by calculating the intrinsic binding constant K_b_ values using Wolfe–Shimmer equation [71] for **3b** and **3c** which was 6.68 × 10^4^ M^−1^ and 1.19 × 10^4^ M^−1^, respectively. The magnitude of the binding constant was lower than those of strong intercalators like EtBr (K_b_ ∼ 10^6^ M^−1^) [72], but comparable to partial intercalators such as ferrocene appended naphthylamide derivatives (K_b_ ∼ 10^4^ M^−1^) [73]. Nevertheless, UV–Vis measurement provides only preliminary information regarding the mode of binding, therefore, further measurement would be required to ensure the binding mode. The spontaneity of the interaction was also assessed by Gibbs free energy change (ΔG) which was − 6.47 and − 5.47 kcal mol^−1^ for compounds **3b** and **3c**, respectively, in which negative values suggest directing of reaction towards product formation.

#### Ethidium bromide displacement measurements

**Fig. 6:**
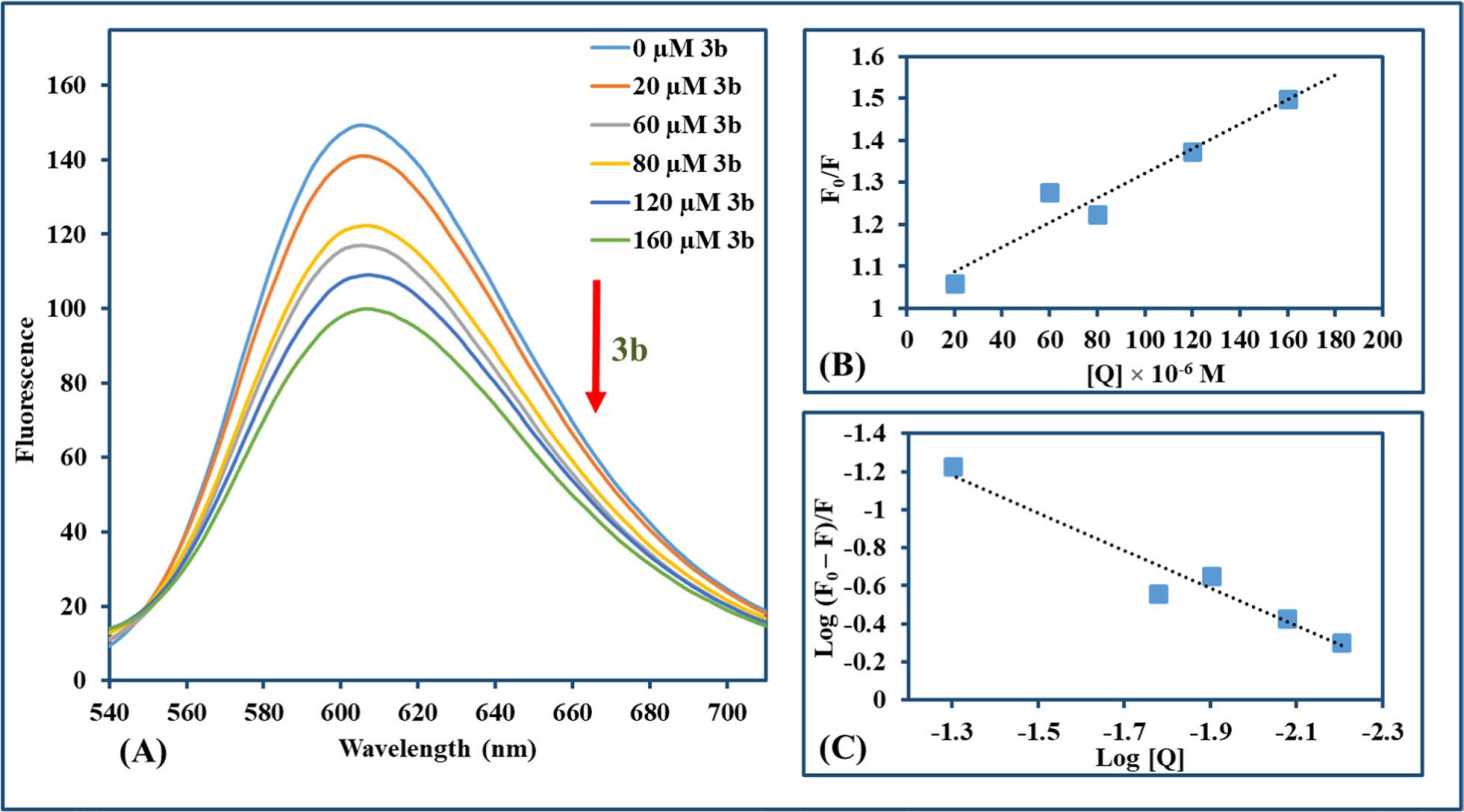
**A** Fluorescence emission spectra of **3b** with CT-DNA-EtBr (λ_ex_ = 500 nm, **3b**: 0–160 µM in the direction of the arrow from lowest to highest). **B** The Stern–Volmer plot quenching effect of **3b** on CT-DNA-EtBr at room temperature. **C** Scatchard method for assigning binding constant K_b_ for **3b** with CT-DNA-EtBr at room temperature

**Fig. 7:**
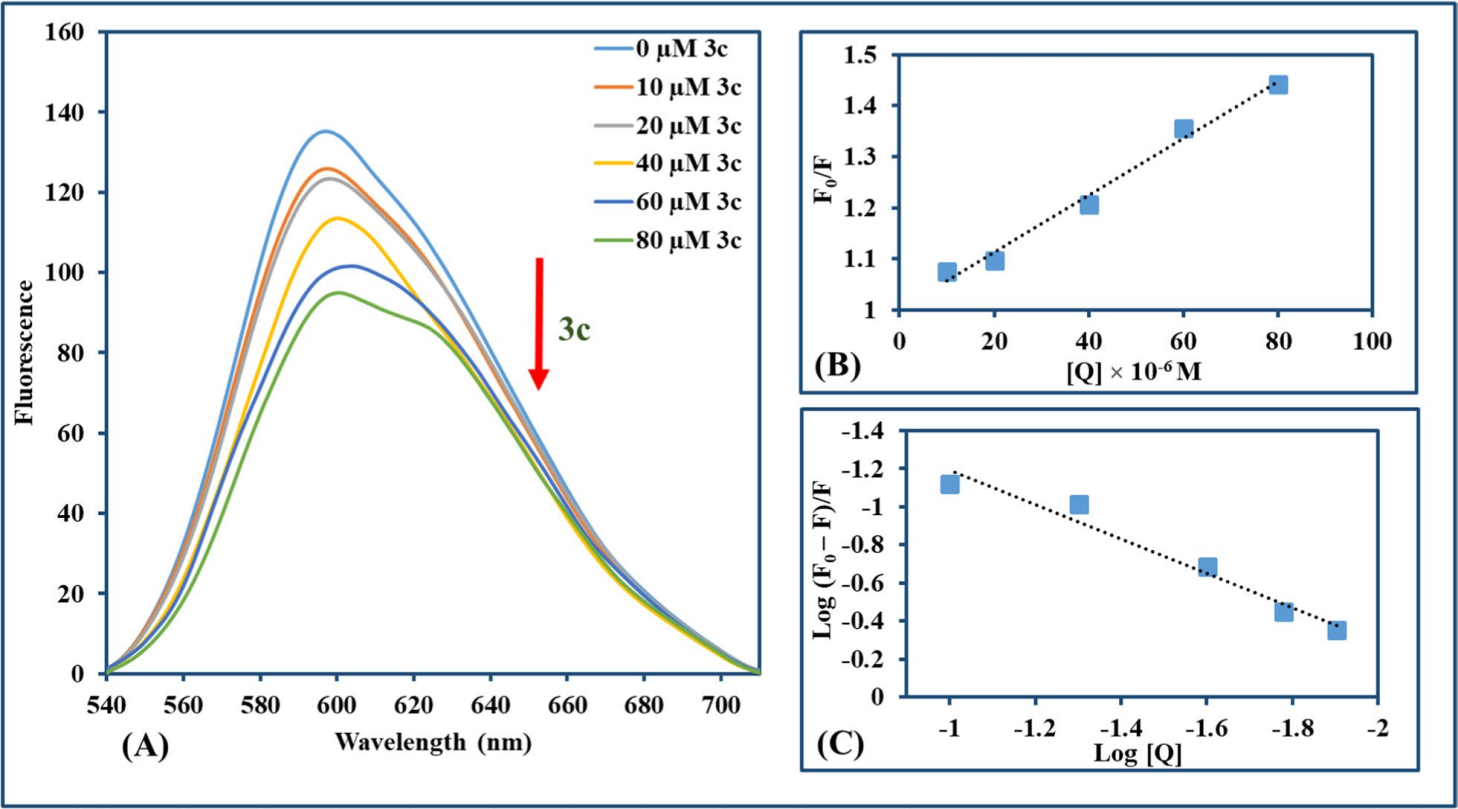
**A** Fluorescence emission spectra of **3c** with CT-DNA-EtBr (λ_ex_ = 500 nm, **3c**: 0–80 µM in the direction of the arrow from lowest to highest). **B** The Stern–Volmer plot quenching effect of **3c** on CT-DNA-EtBr at room temperature. **C** Scatchard method for assigning binding constant K_b_ for **3c** with CT-DNA-EtBr at room temperature

The interaction mode between compound and CT-DNA can be discriminated through fluorescence spectroscopy with the EtBr–CT-DNA system [74]. EtBr is a classic indicator of intercalation where an intense fluorescence signal is emitted from the EtBr-CT-DNA system through intercalation of EtBr between adjacent DNA base pairs with no obvious signal for unaccompanied EtBr [75, 76]. According to earlier research, substances with comparable DNA binding modes can substitute intercalated EtBr, significantly reducing EtBr's fluorescence intensity [77]. In order to demonstrate the fluorescence emission spectra of EtBr-CT-DNA at an excitation wavelength of 500 nm, fluorescence titration for each compound (**3b** and **3c**) against EtBr-CT-DNA was carried out. This accomplished a significant reduction in fluorescence intensity (~ 33.3% and ~ 30.7%, respectively) in the presence of **3b** and **3c**, as illustrated in Figs. 6 and 7, respectively. The Stern–Volmer equation was used for the study of the effect of quenchers on the CT-DNA-EtBr system [78, 79].

$${\text{F}}_{0}/{\text{F}}{\text{= 1+} }{\text{K}}_{\text{sv}}\left[{\text{Q}}\right]{\text{ = 1+} }{\text{k}}_{\text{q}}{\tau }_{0}{\text{ [Q] }}$$

where F_0_ and F are fluorescence intensities in the absence and presence of the compound used for quenching, respectively; K_SV_ is a linear Stern–Volmer quenching constant; [Q] is the concentration of the quenching compound and τ_0_ is the fluorescence lifetime of the fluorophore (10^−8^ s) in absence of the compound responsible for quenching. K_SV_ for **3b** and **3c** is computed from the linear regression plot of F_0_/F against [Q]. Also, the binding constant (K_b_) and the number of binding sites (n) were determined by the Scatchard method [80].

$${\text{log }}(({{\text{F}}_{0}}-{\text{F}})/{\text{F}})={\text{log}}{{\text{ K}}_{\text{b}}}+ {\text{n}} {\text{ log}}{\text{ [Q]}}$$

K_b_ has been assigned as the intercept and n as the slope in the linear plotting of log((F_0_–F)/F) versus log[Q]. The detailed results for **3b** and **3c** were computed and depicted in Table 1. It was found that K_b_ values obtained for **3b** and **3c** were very small in comparison to the reported value of a classical intercalator of EtBr–DNA which is in order of 10^7^ M^−1^ [81]. Moreover, **ΔG** for both **3b** and **3c** showed negative values indicating that the interaction is spontaneous.

**Table 1:** Parameters of **3b** and **3c** interaction with CT-DNA-EtBr: quenching constants (K_SV_), quenching rate constant (k_q_), binding constants (K_b_), binding site numbers (n), and binding energy (ΔG)

Compound	K_SV_ (M^−1^)	R^2^ (B)	k_q_ (M^−1^ s^−1^)	K_b_ (M^−1^)	R^2^ (C)	n	(ΔG) (kJ mol^–1^)
3b	2.9 × 10^3^	0.930	2.9 × 10^11^	2.93 × 10^2^	0.942	0.99	− 0.27
3c	5.6 × 10^3^	0.987	5.6 × 10^11^	1.24 × 10^2^	0.961	0.90	− 0.31

### BSA binding studies

#### UV–Visible measurements

**Fig. 8:**
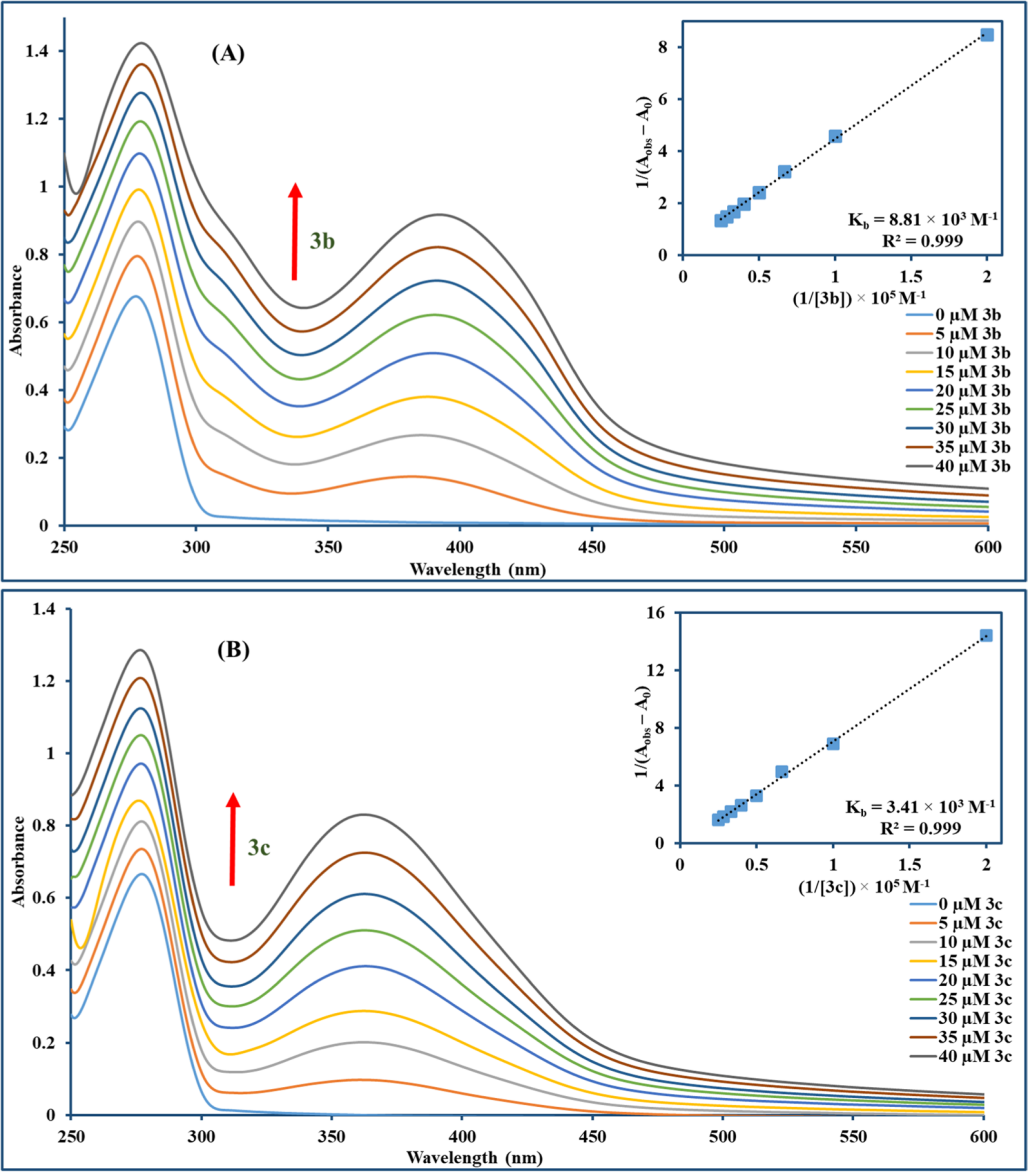
UV–Vis absorption spectra of BSA (15 µM) in the absence and presence of **3b** (**A**) and **3c** (**B**), showed that the increase in the concentration of compounds (0–40 µM) had a hyperchromic effect denoted by the arrow direction. The incorporated graph exemplified the plot used for binding constant (K_b_) computation

Several investigations undoubtedly focus on serum albumin owing to its prominence and abundance in blood plasma [82]. Besides its crucial role in the exogenous and endogenous molecules transportation, the binding ability of albumin to small molecules as drugs enhances solubility, and drug-life time, and reduces their toxicity, which is functional in therapeutic targets [83]. Moreover, it has a role in the regulation of redox potential and colloid osmotic pressure between blood and tissue [84]. Therefore, albumins have extensive physiological, medical, and biochemical applications [85]. BSA has opted for investigation concerning bioactive chemical binding and homology to human serum albumin [86]. UV–Vis spectrophotometric titration effectively investigated the physical changes in proteins and analysed the protein–drug interaction [87]. The interaction between compounds (**3b** and **3c**) and BSA was examined (Fig. 8), revealing that **3b** and **3c** had noteworthy hyperchromicity (~ 52.2% and ~ 48.2%) at 277 nm, respectively. Furthermore, the estimated K_b_ values for **3b** and **3c** were found to be 8.81 × 10^3^ and 3.41 × 10^3^ M^−1^, respectively. The interaction capability was also predicted by computation of ΔG that was shown to be − 7.6 for **3b** and − 6.8 kcal mol^−1^ for **3c**, where negative values imply the spontaneous interaction between the compounds and BSA.

#### Tryptophan quenching experiment

**Fig. 9:**
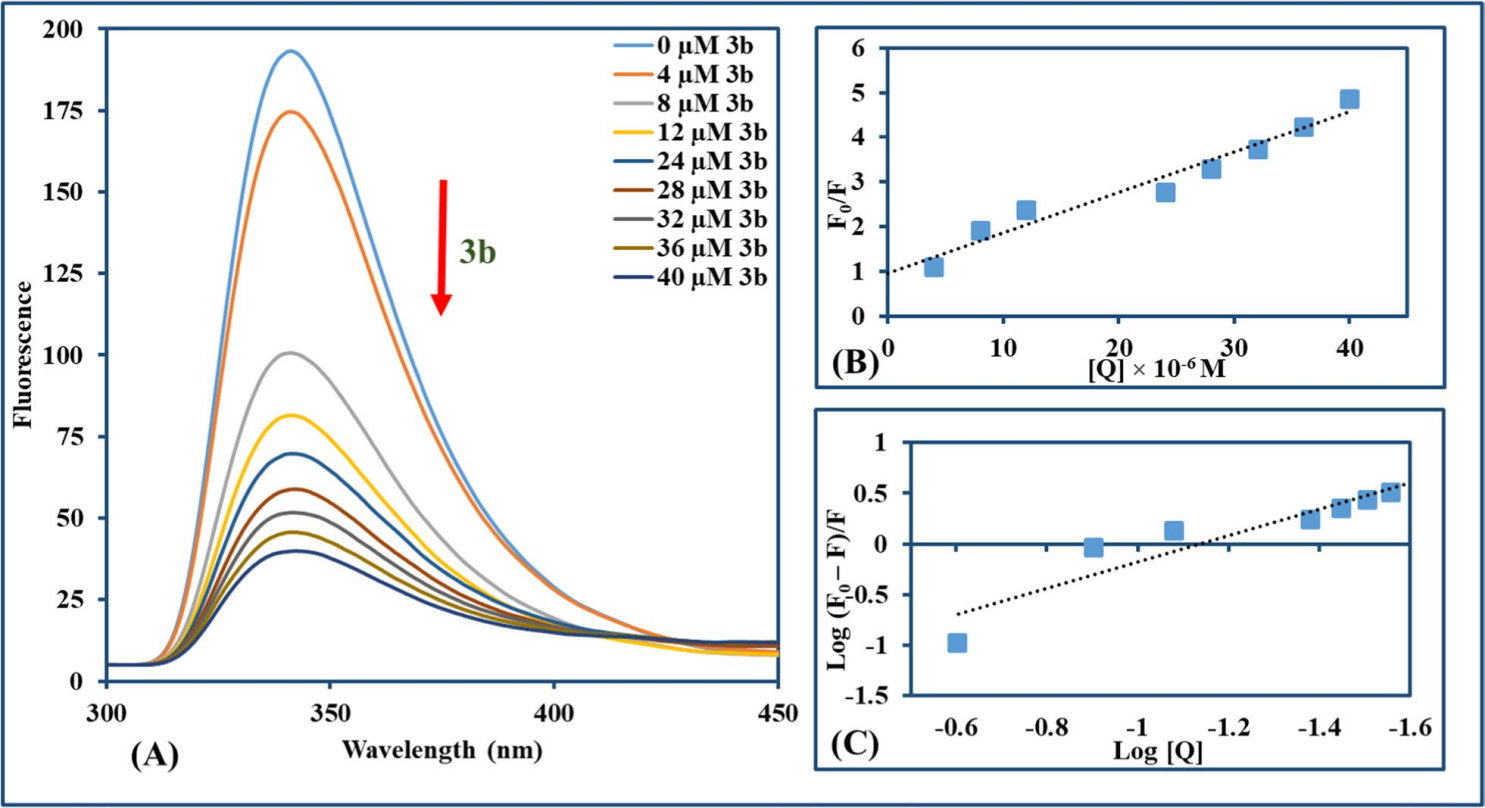
**A** Fluorescence emission spectra of BSA (15 μM) with increasing amounts of **3b** (0–40 μM) (λ_ex_ = 289 nm, with the arrow direction from the lowest to the highest concentration of **3b**). **B** The Stern–Volmer plot quenching effect of **3b** on BSA at room temperature. **C** Scatchard method for assigning K_b_ for BSA with **3b** at room temperature

**Fig. 10:**
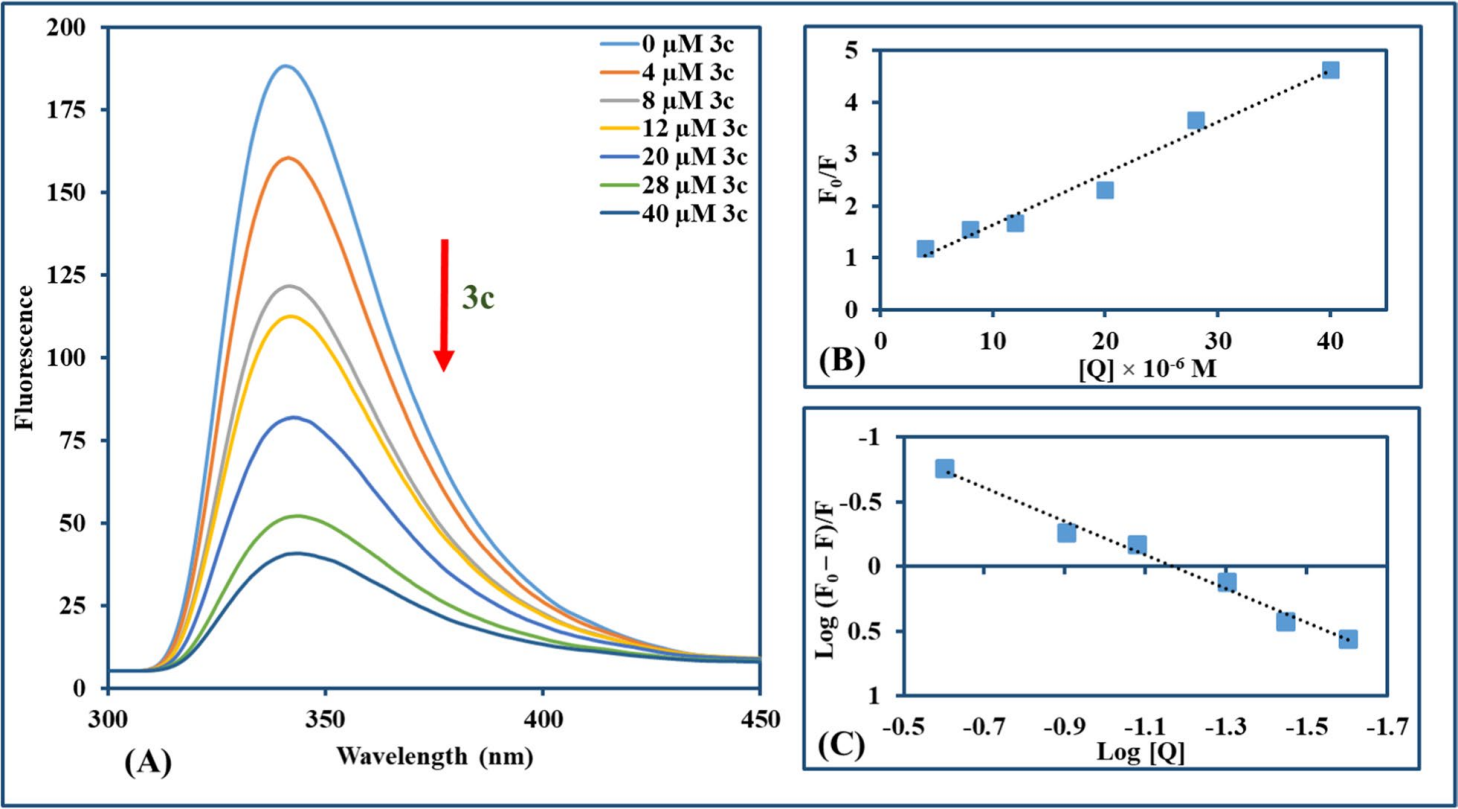
**A** Fluorescence emission spectra of BSA (15 μM) with increasing amounts of **3c** (0–40 μM) (λ_ex_ = 289 nm, with the arrow direction from the lowest to the highest concentration of **3c**). **B** The Stern–Volmer plot quenching effect of **3c** on BSA at room temperature. **C** Scatchard method for assigning K_b_ for BSA with **3c** at room temperature

Fluorescence investigations could be used to conduct a specified analysis of whether synthetic molecules bind to biomolecules as BSA. It is widely perceived that predominantly tryptophan (Trp) is responsible for the inherited fluorescence among the other two amino acids [tyrosine (Tyr) and phenylalanine (Phe)] in the BSA [88]. The tryptophan emission spectrum is frequently altered because of protein conformational variation, subunit interactions, substrate binding, or denaturation [89]. Reduced fluorescence emission intensity is observed for **3b** and **3c**, which may be due to disrupted BSA structure altering the tryptophan microenvironments. Moreover, the internalization of fluorophore residues into the protein's hydrophobic core is due to protein folding or related to the hydrophobic amino acid residues around Trp, such as Leu, Tyr, and phe [90]. The serial addition of the **3b** and **3c** to the BSA solution revealed a reduction in the maximum peak emission of BSA, which was around 341 nm, as depicted in Figs. 9 and 10. Also, a linear plot of F_0_/F against the concentration of **3b** and **3c** was used for computing the values of K_SV_ (9.0 × 10^4^ and 9.9 × 10^4^ M^−1^) and k_q_ (9.0 × 10^12^ and 9.9 × 10^12^ M^−1^ s^−1^) for the titled compounds (**3b** and **3c**, respectively**)**. Furthermore, the K_b_ value (~ 0.3 × 10^2^ and 0.34 × 10^2^ M^−1^) and n value (~ 1.3) assessed from the plot of log(F_0_−F/F) against log(compound concentration) was nearly the same for both **3b** and **3c**. The negative values of ΔG also supported the UV–Vis results of free energy changes and implied the spontaneity of **3b**/**3c**–BSA binding, where the interaction parameters were illustrated in Table 2.

**Table 2:** Parameters of **3b** and **3c** interaction with BSA: quenching constants (K_SV_), quenching rate constant (k_q_), binding constants (K_b_), binding site numbers (n), and binding energy (ΔG)

Compound	K_SV_ (M^−1^)	R^2^ (B)	k_q_ (M^−1^ s^−1^)	K_b_ (M^−1^)	R^2^ (C)	n	(ΔG) (kJ mol^–1^)
3b	9.0 × 10^4^	0.956	9.0 × 10^12^	0.30 × 10^2^	0.884	1.3	− 2.02
3c	9.9 × 10^4^	0.977	9.9 × 10^12^	0.34 × 10^2^	0.985	1.3	− 2.08

### Cytotoxic studies

**Fig. 11:**
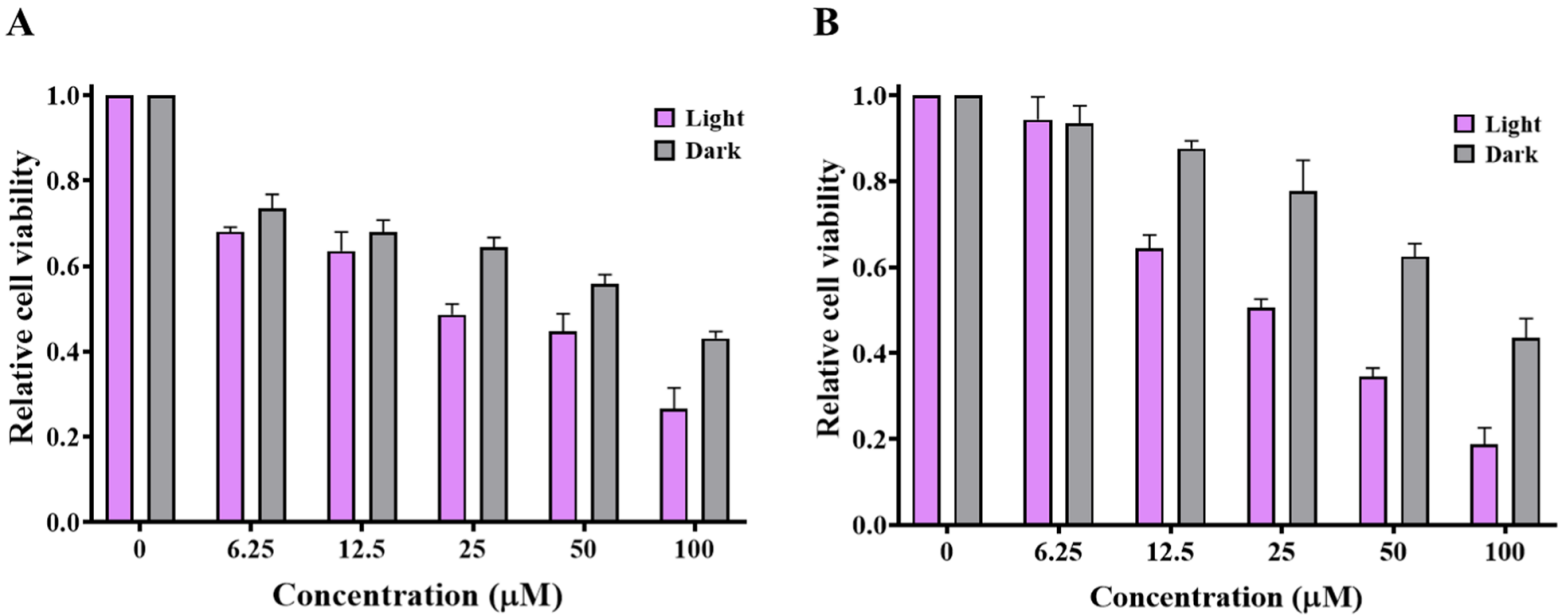
The cytotoxic effects of **3b** (**A**) and **3c** (**B**) on human colon cancer (HCT116) cells without/with illumination at 365 nm for 20 min at different concentrations (0, 6.25, 12.5, 25, 50 and 100 µM)

To assess the cytotoxic impact of compounds **3b** and **3c** on HCT116 and MDA-MB-231 cancer cells with and without illumination, the MTT test was conducted. As shown in (Fig. 11), a slight cytotoxic effect of compounds **3b** and **3c** on the HCT116 cell line in the absence of illumination with IC_50_ values of 72 and 82 µM, respectively, compared to doxorubicin as a positive control (Fig. S11), with IC_50_ value of 40 µM. A significant reduction in viability was observable upon irradiation with substantial IC_50_ values of 23 and 25 µM for compounds **3b** and **3c**, respectively.

**Fig. 12:**
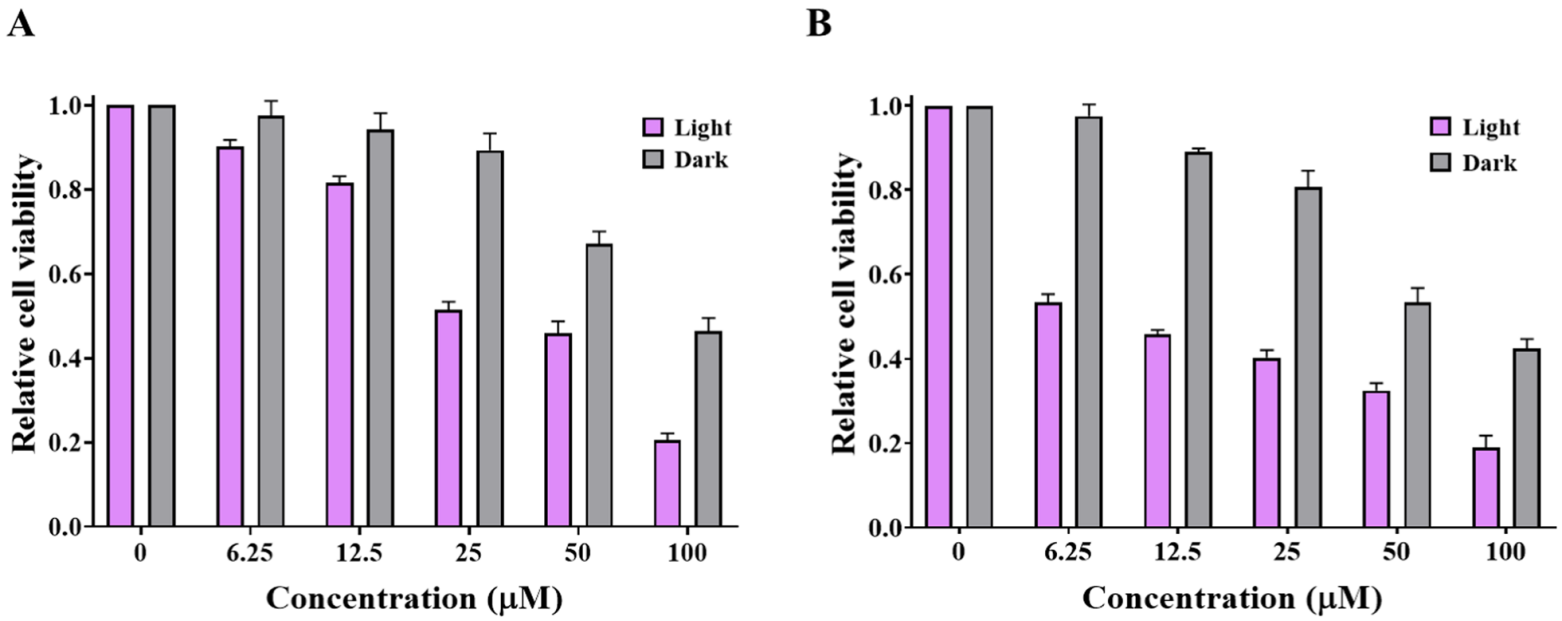
The cytotoxic effects of **3b **(**A**) and **3c** (**B**) on human breast cancer (MDA-MB-231) cells without/with illumination at 365 nm for 20 min at different concentrations (0, 6.25, 12.5, 25, 50 and 100 µM)

Furthermore, **3b** and **3c** revealed a weaker anticancer potential against MDA-MB-231 cells (Fig. 12) in the dark conditions with IC_50_ values of 91 and 64 µM, respectively, relative to doxorubicin with IC_50_ value of 64.8 µM (Fig. S11). On the other hand, treating MDA-MB-231 cells with the titled compounds under illuminating conditions (Fig. 12) revealed remarkable IC_50_ values of 30 and 9 µM for compounds **3b** and **3c**, respectively. This may be accredited to free radicals’ species generation as singlet oxygen and superoxide, which facilitate the process of apoptosis for cancer cells.

## Conclusion

A novel six thiazole-related cyanoacrylamide derivatives (**3a**–**f**) were synthesized and characterized and their nuclease activity was assessed through DNA cleavage experiment. These derivatives exhibited insignificant chemically induced nuclease activity but on irradiation, this activity was improved mostly for the derivatives **3b** and **3c** through the released singlet oxygen and superoxide free radical species. Moreover, a partial intercalation binding of **3b** and **3c** with CT-DNA was supported by ethidium bromide displacement and UV–Vis measurements. In addition, **3b** and **3c** interactions with BSA, demonstrated by tryptophan quenching and UV–Vis measurements, could provide facilitated movement inside the human body. The evaluation of the cytotoxic effect against colon and breast cancer cells, before and after irradiation revealed an enhanced activity that could serve as a base for the synthesis of potential cancer therapeutic agents that specifically function with their enhanced effect under irradiation. Therefore, targeting this advantageous property toward other biological activities may reveal promising results. In general, photodynamic therapy for cancer treatment could benefit from these thiazole-related cyanoacrylamide derivatives (**3b** and **3c**) for targeted therapy that depends on irradiation.

## Supplementary Information

Supplementary Material 1

## Data Availability

Data available at request (Dr. Mohamed A. Ragheb, and Ismail A. Abdelhamid, email: mattia@cu.edu.eg; ismail_shafy@cu.edu.eg; ismail_shafy@yahoo.com). All data generated or analyzed during this study are included in this published article and its supplementary information file.
