# Supplementary material for: Novel thiazole-based cyanoacrylamide derivatives: DNA cleavage, DNA/BSA binding properties and their anticancer behaviour against colon and breast cancer cells
Source: BMC Chem. 2024 Sep 20;18(1):183. doi: 10.1186/s13065-024-01284-2 (PMC11414077; doi:10.1186/s13065-024-01284-2)
Supplement: Supplementary file 1 — Supplementary Material 1 [file 13065_2024_1284_MOESM1_ESM.docx]

**Supplementary Data**

**Novel Thiazole-Based Cyanoacrylamide Derivatives: DNA cleavage, DNA/BSA binding properties and their anticancer Behaviour against colon and breast cancer cells**

**The NMR spectra of new compounds**

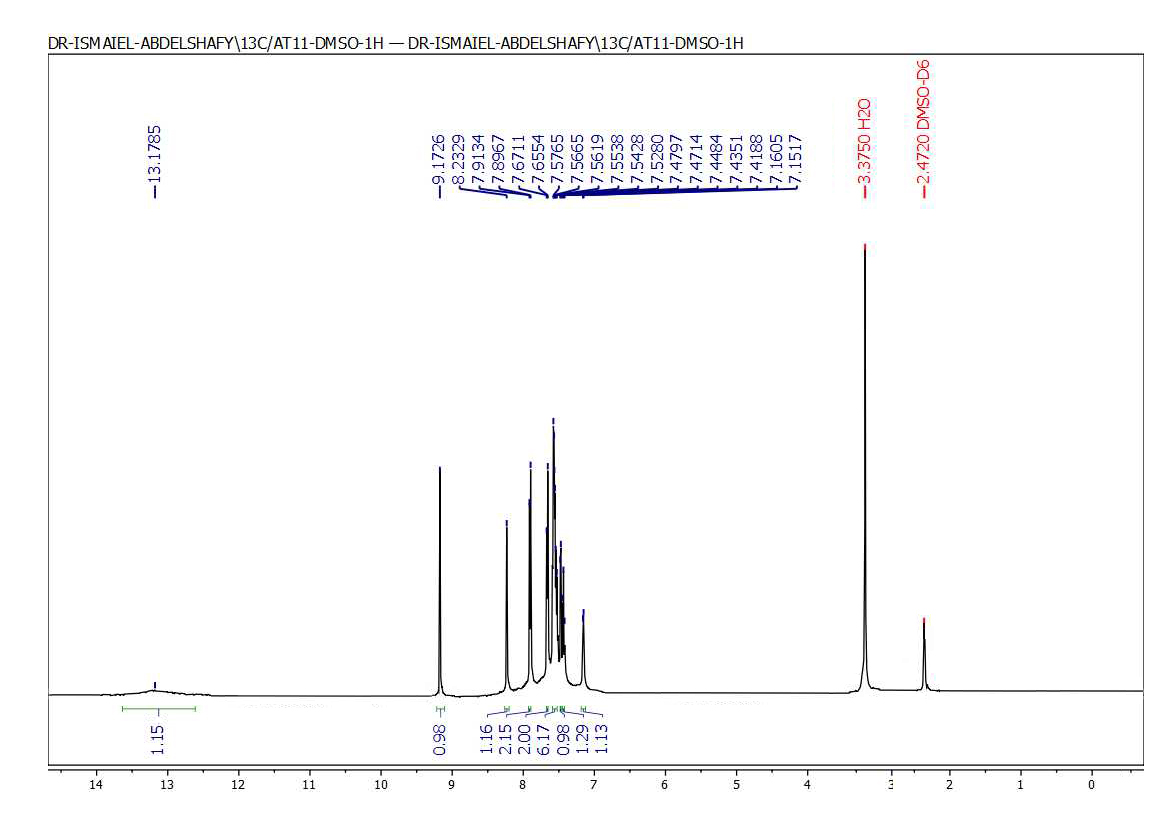


**Figure S1.** The ^1^H NMR spectrum of compound **3a**

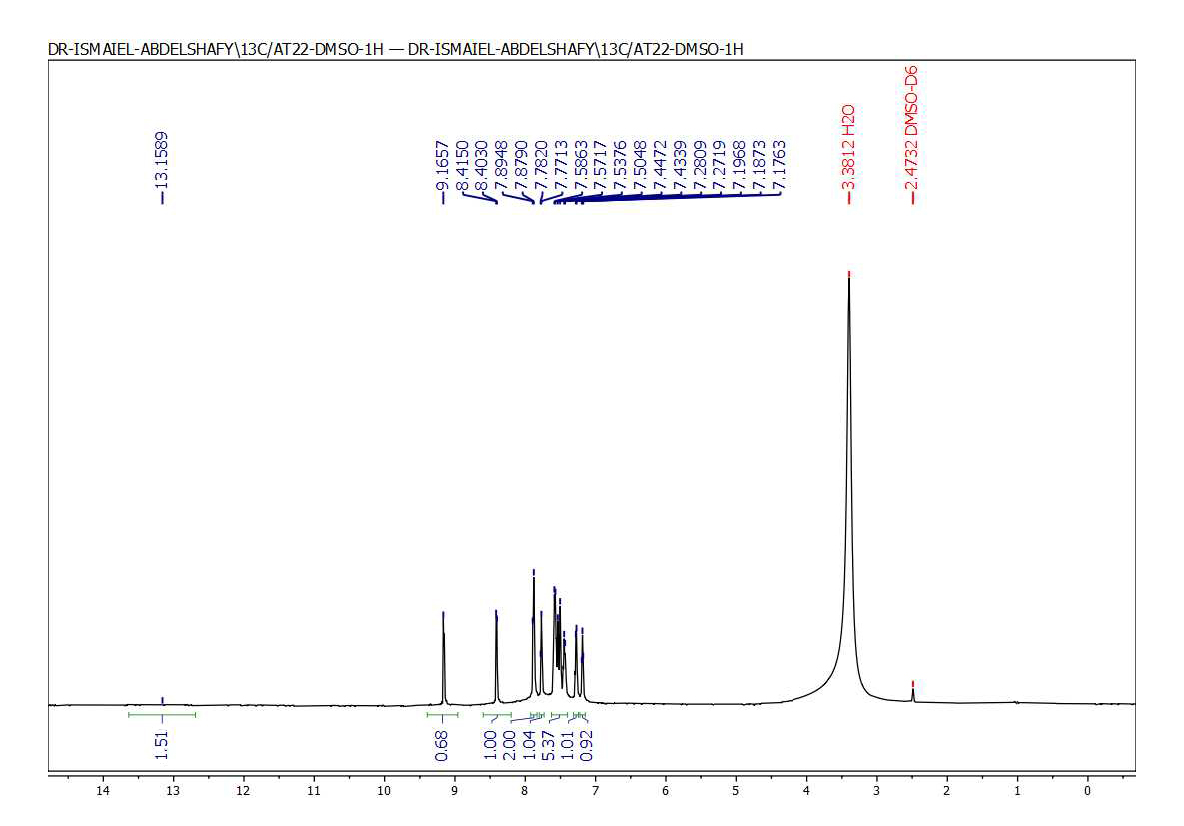


**Figure S2.** The ^1^H NMR spectrum of compound **3b**

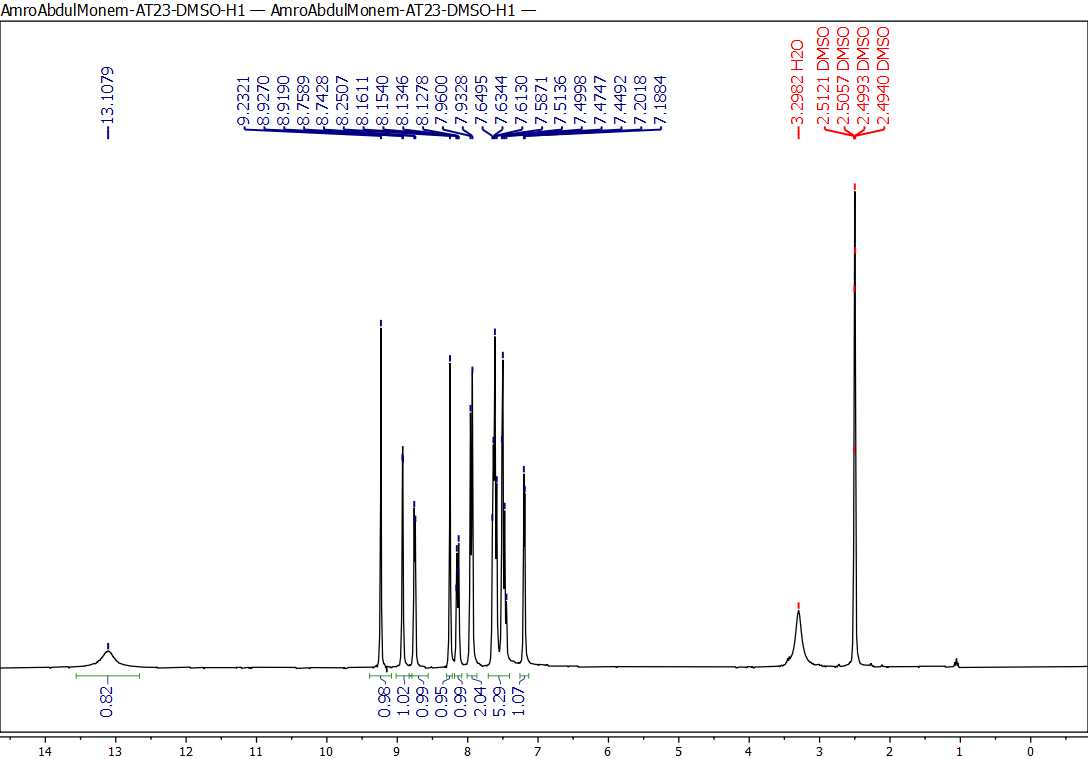


**Figure S3.** The ^1^H NMR spectrum of compound **3c**

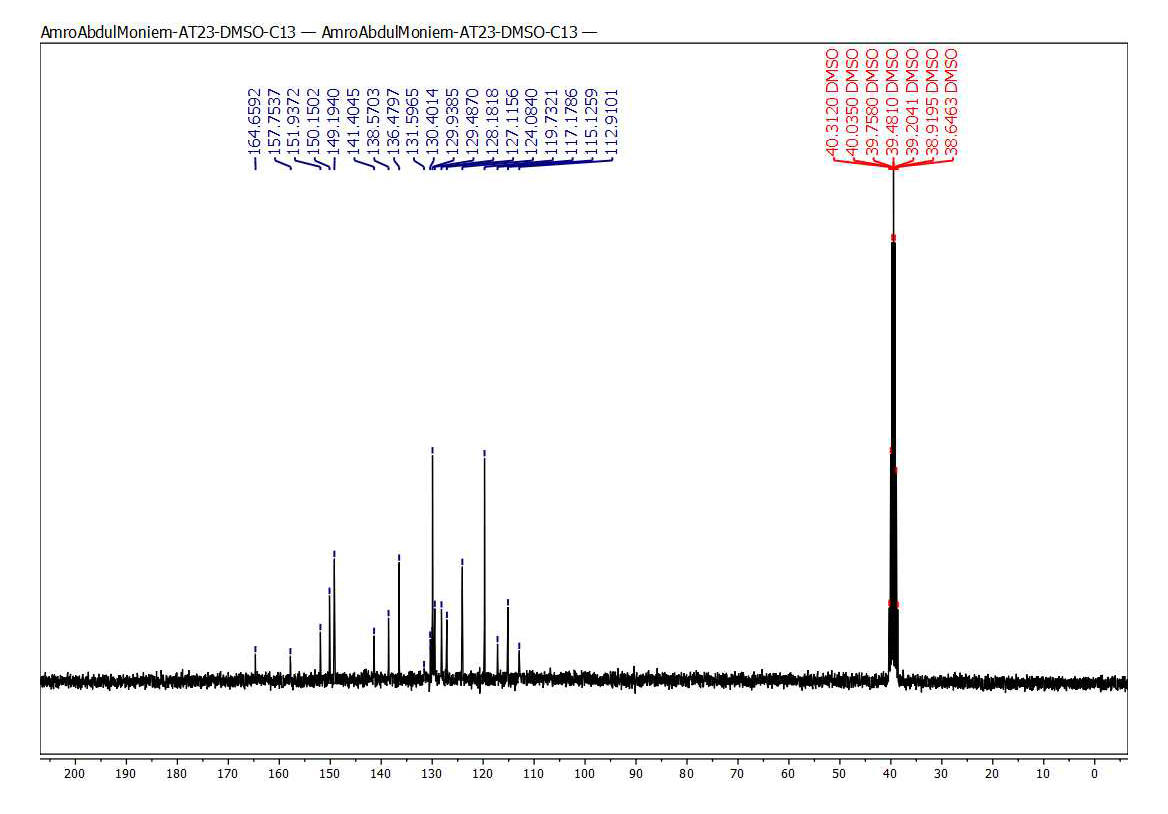


**Figure S4.** The ^13^C NMR spectrum of compound **3c**

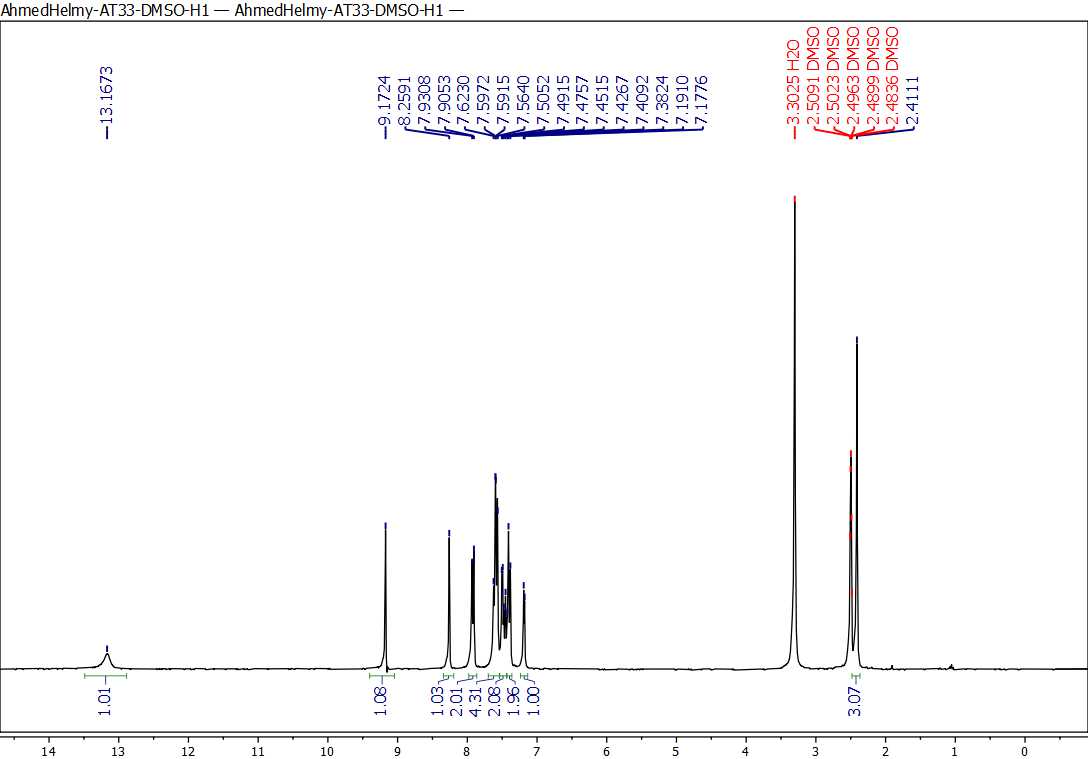


**Figure S5.** The ^1^H NMR spectrum of compound **3d**

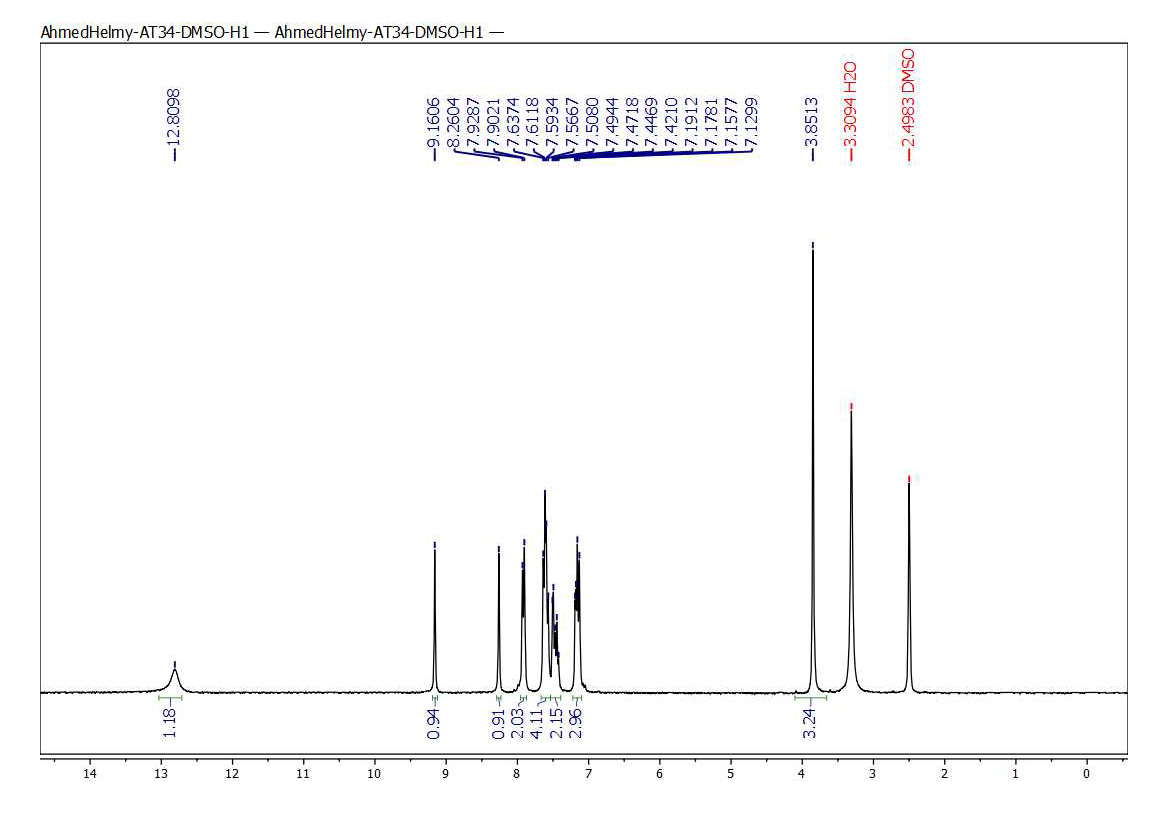


**Figure S6.** The ^1^H NMR spectrum of compound **3e**

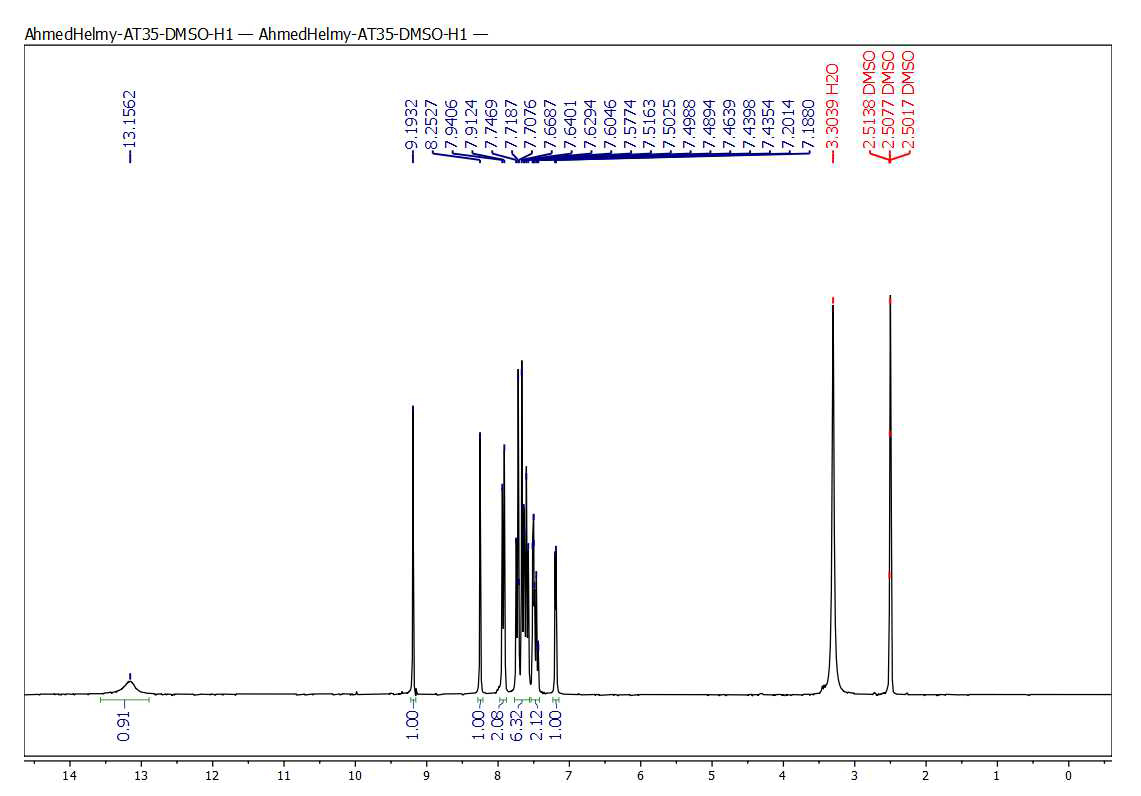


**Figure S7.** The ^1^H NMR spectrum of compound **3f**

**
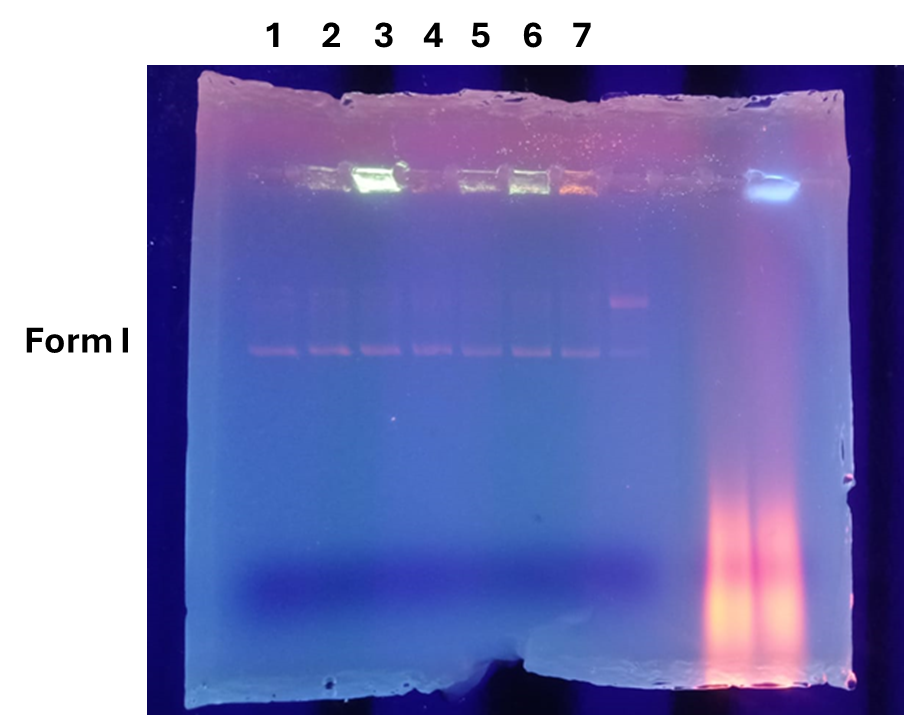
**

**Figure S8.** Agarose gel electrophoresis pattern of pBR322 DNA (0.3 μg) cleavage by compounds 3a-f (200μM) incubated in the dark at 37 °C for 30 min. Lane 1: DNA control; Lane 2: DNA+3a; Lane 3: DNA +3b; Lane 4: DNA+ 3c; Lane 5; DNA+3d; Lane 6: DNA+3e; Lane 7: DNA+3f.


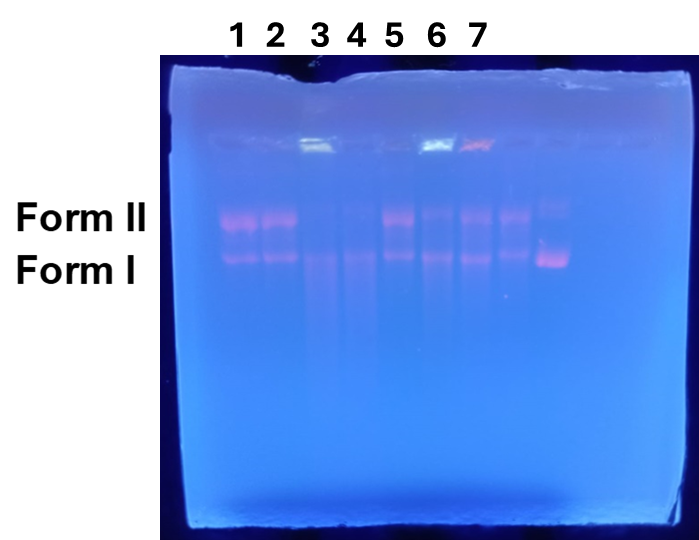


**Figure S9.** Agarose gel electrophoresis pattern of pBR322 DNA (0.3 μg) cleavage by compounds **3a-f** (200μM) incubated in the dark at 37 °C for 30 min followed by irradiation at 365 nm for 15 min. Lane 1: DNA control; Lane 2: DNA+**3a**; Lane 3: DNA +**3b**; Lane 4: DNA+ **3c**; Lane 5; DNA+**3d**; Lane 6: DNA+**3e**; Lane 7: DNA+**3f**.


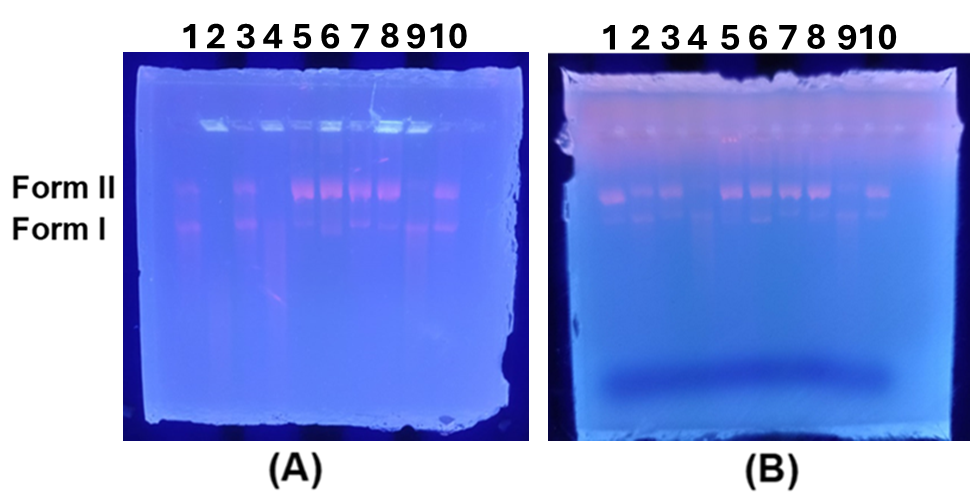


**Figure S10.** Agarose gel electrophoresis pattern of pBR322 DNA (0.3 μg) cleavage by **3b** (200μM) **(A)** and **3c** (200μM) **(B)** with different free radical scavengers incubated in the dark at 37 °C for 30 min followed by irradiation at 365 nm for 15 mi. DNA. Lane 3: DNA+DMSO (200mM); Lane 4: DNA+**CPD**+DMSO (200Mm); Lane 5: DNA+KI (200mM); Lane 6: DNA+**CPD**+KI (200mM); Lane 7; DNA+NaN_3_ (200mM); Lane 8: DNA+**CPD**+NaN_3_ (200mM); Lane 9: DNA+**CPD**; Lane 10: DNA control.

**Figure S11.** The cytotoxic effects of doxorubicin on human colon cancer (HCT116) and human breast cancer (MDA-MB-231) cells at different concentrations (0, 6.25, 12.5, 25, 50 and 100 µM).


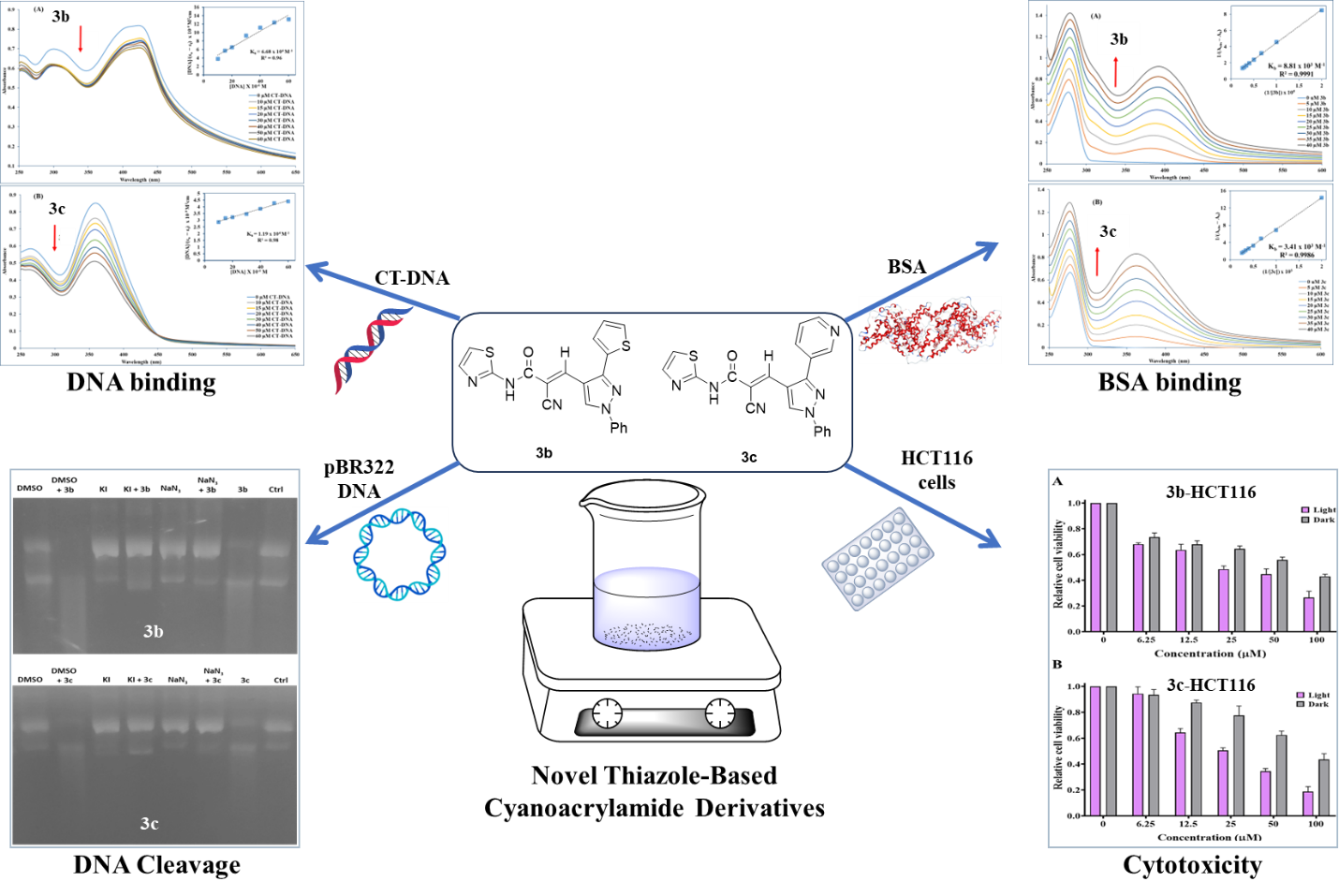


**Figure S12**. Novel thiazole-based cyanoacrylamide derivatives (**3b** and **3c**) revealed significant CT-DNA/BSA binding affinities, pBR322 plasmid DNA photocleavage activities, and cytotoxic efficacy against HCT116 colon cancer cells.
